# Curcumin synergistically enhances the efficacy of gemcitabine against gemcitabine-resistant cholangiocarcinoma via the targeting LAT2/glutamine pathway

**DOI:** 10.1038/s41598-024-66945-7

**Published:** 2024-07-11

**Authors:** Phonpilas Thongpon, Kitti Intuyod, Sasitorn Chomwong, Thatsanapong Pongking, Sirinapha Klungsaeng, Kanha Muisuk, Naruechar Charoenram, Chutima Sitthirach, Raynoo Thanan, Porntip Pinlaor, Somchai Pinlaor

**Affiliations:** 1 Department of Parasitology, Faculty of Medicine, Khon Kaen University, Khon Kaen, 40002 Thailand; 2 Department of Pathology, Faculty of Medicine, Khon Kaen University, Khon Kaen, 40002 Thailand; 3 Biomedical Sciences Program, Graduate School, Khon Kaen University, Khon Kaen, 40002 Thailand; 4 Department of Forensic Medicine, Faculty of Medicine, Khon Kaen University, Khon Kaen, 40002 Thailand; 5 Department of Biochemistry, Faculty of Medicine, Khon Kaen University, Khon Kaen, 40002 Thailand; 6 Centre for Research and Development in Medical Diagnostic Laboratory, Faculty of Associated Medical Sciences, Khon Kaen University, Khon Kaen, 40002 Thailand; 7 Cholangiocarcinoma Research Institute, Khon Kaen University, Khon Kaen, 40002 Thailand

**Keywords:** Cholangiocarcinoma, Curcumin, Gemcitabine, Drug resistance, *SLC7A8* (LAT2), Glutamine, Cancer, Cell biology, Molecular biology, Diseases

## Abstract

Cholangiocarcinoma (CCA) is often diagnosed late, leading to incomplete tumor removal, drug resistance and reduced chemotherapy efficacy. Curcumin has the potential for anti-cancer activity through various therapeutic properties and can improve the efficacy of chemotherapy. We aimed to investigate the synergistic effect of a combination of curcumin and gemcitabine against CCA, targeting the LAT2/glutamine pathway. This combination synergistically suppressed proliferation in gemcitabine-resistant CCA cells (KKU-213B^GemR^). It also resulted in a remarkable degree of CCA cell apoptosis and cell cycle arrest, characterized by a high proportion of cells in the S and G2/M phases. Knockdown of *SLC7A8* decreased the expressions of glutaminase and glutamine synthetase, resulting in inhibited cell proliferation and sensitized CCA cells to gemcitabine treatment. Moreover, in vivo experiments showed that a combination curcumin and gemcitabine significantly reduced tumor size, tumor growth rate and LAT2 expression in a gemcitabine-resistant CCA xenograft mouse model. Suppression of tumor progression in an orthotopic CCA hamster model provided strong support for clinical application. In conclusion, curcumin synergistically enhances gemcitabine efficacy against gemcitabine-resistant CCA by induction of apoptosis, partly via inhibiting LAT2/glutamine pathway. This approach may be an alternative strategy for the treatment of gemcitabine-resistant in CCA patients.

## Introduction

Cholangiocarcinoma (CCA) is a bile-duct cancer. Its incidence rate is increasing globally and currently accounts for ~ 10–15% of all primary liver cancers^1^. The highest incidence of CCA is found in Thailand, particularly in the northeastern region, where 85 cases per 100,000 persons per year were reported. During the years 2009 to 2013, the one-year mortality rate in Thailand exceeded 80%^2^. CCA is an aggressive tumor with poor prognosis, and previous nonsurgical curative therapies have been markedly ineffective^3^. Infestation with liver fluke (*Opisthorchis viverrini* and *Clonorchis sinensis)* is one of the most important risk factors for CCA in the Mekong Basin subregion. In particular, *O. viverrini* is a major public-health problem in Thailand and neighboring countries^4^.

Currently, surgical resection of the tumor is the conventional treatment option, but most CCA patients are diagnosed at an advanced stage and removal of the tumor is often incomplete. Remaining options, such as conventional chemotherapy and radiotherapy, are ineffective due to drug resistance. Thus, new effective chemopreventive and adjuvant therapeutic strategies for CCA are needed^5^. The most popular drug used for chemotherapy of CCA is gemcitabine. However, by the time most CCA patients are diagnosed, the tumor is resistant to chemotherapy^6^. Hence, an alternative treatment that focuses on molecules that confer drug resistance in CCA could have potential application.

Curcumin, (diferuloylmethane), a natural product extracted from the rhizomes of turmeric (*Curcuma longa*), has anti-oxidant, anti-inflammatory, anti-microbial, anti-fibrotic and anti-cancer activities. Curcumin is useful in the prevention and treatment of various diseases such as cancer, autoimmune diseases, neurological diseases, cardiovascular diseases and diabetes^7,8^. Curcumin exerts its therapeutic effects by suppressing the activation of oncogenic transcription factors, anti-apoptotic proteins, and cell cycle proteins, and can induce the expression of pro-apoptotic proteins^9^. Curcumin is being tested for use as an alternative anti-CCA agent^10,11^. It also has the potential to improve the sensitivity of chemotherapy in drug-resistance models in various cancer types including pancreatic cancer^12^, bladder cancer^13^ and colon cancer^14^.

The *SLC7A8* gene encodes a neutral amino-acid transporter protein called LAT2 (L-type amino-acid transporter-2). LAT2 plays a critical role for the uptake of neutral amino acids, including glutamine, which are essential nutrients for the growth and proliferation of cancer cells, and are crucial to the biosynthesis of products such as nucleic acids and non-essential amino acids ^15^. According to data from Oncomine, LAT2 is upregulated in nine different cancers including breast cancer, colorectal cancer, lymphoma and leukemia^16^. Recent research has shown that LAT2 is upregulated in invasive breast cancer^17^ and gemcitabine-resistant pancreatic cancer cells^18^. This protein serves as an oncogenic factor and regulates mammalian target of rapamycin activation in a glutamine-dependent manner, promoting glycolysis and reducing the sensitivity of pancreatic cancer cells to gemcitabine^18^. However, the role of LAT2/glutamine in CCA, especially in the cortex of gemcitabine-resistance has not yet been elucidated. Furthermore, whether anti-CCA activities as well as improvement of gemcitabine efficacy are partly mediated through modulation of LAT2 expression is unknown.

In this study, we aimed to investigate the role of LAT2/glutamine in gemcitabine resistance and the therapeutic potential of curcumin, especially for improvement of gemcitabine treatment using both in vitro and in vivo models of gemcitabine-resistant CCA. The results of the study can inform future clinical application.

## Results

### Establishment and characterization of drug-resistant (KKU-213B^GemR^)

**Figure 1:**
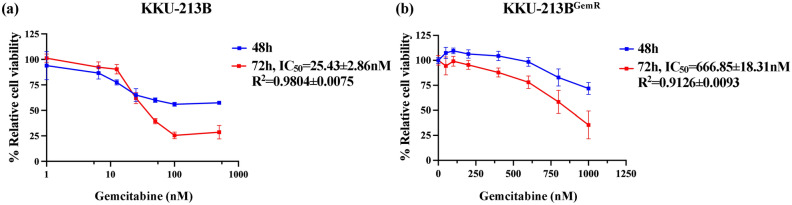
Half-maximal inhibitory concentration (IC_50_) in CCA cell lines incubated for 48 h or 72 h with the indicated concentration of gemcitabine. (**a**) Parental CCA cells (KKU-213B); 0–500 nM and (**b**) Gemcitabine-resistant CCA cells; 0–1,000 nM. All data are expressed as mean ± SD from biological and technical replicate experiments.

To verify whether the KKU-213B^GemR^ cells were derived from the parental KKU-213B cell line, the short tandem repeat (STR) profiles of the parental (KKU-213B) and gemcitabine-resistant CCA cell lines (KKU-213B^GemR^) were compared. The result showed that the STR profiles of KKU-213B and KKU-213B^GemR^ were identical, both in X-chromosome STR and autosomal STR profiles (Supplementary data Table S1). The half-maximal inhibitory concentration (IC_50_) after 40 cycles of gemcitabine-resistance induction was far higher (IC_50_, 139.85 ± 12.23 nM) for KKU-213B^GemR^ than for parental KKU-213B (IC_50_, 25.43 ± 2.86 nM). The IC_50_ value was therefore 5.50-fold higher in KKU-213B^GemR^ than in KKU-213B cells, with a correlation coefficient (R^2^) above 0.9 (Supplementary data Fig. S1, Fig. 1a). After 60 cycles, the IC_50_ of KKU-213B^GemR^ increased to 26.22-fold (IC_50_, 666.85 ± 18.31 nM) with a correlation coefficient (R^2^) greater than 0.9 (Fig. 1b). These findings imply that extended and repeated gemcitabine exposure increases the KKU-213B cells’ resistance to the drug.

### Curcumin and gemcitabine suppress cell proliferation and colony formation

**Figure 2:**
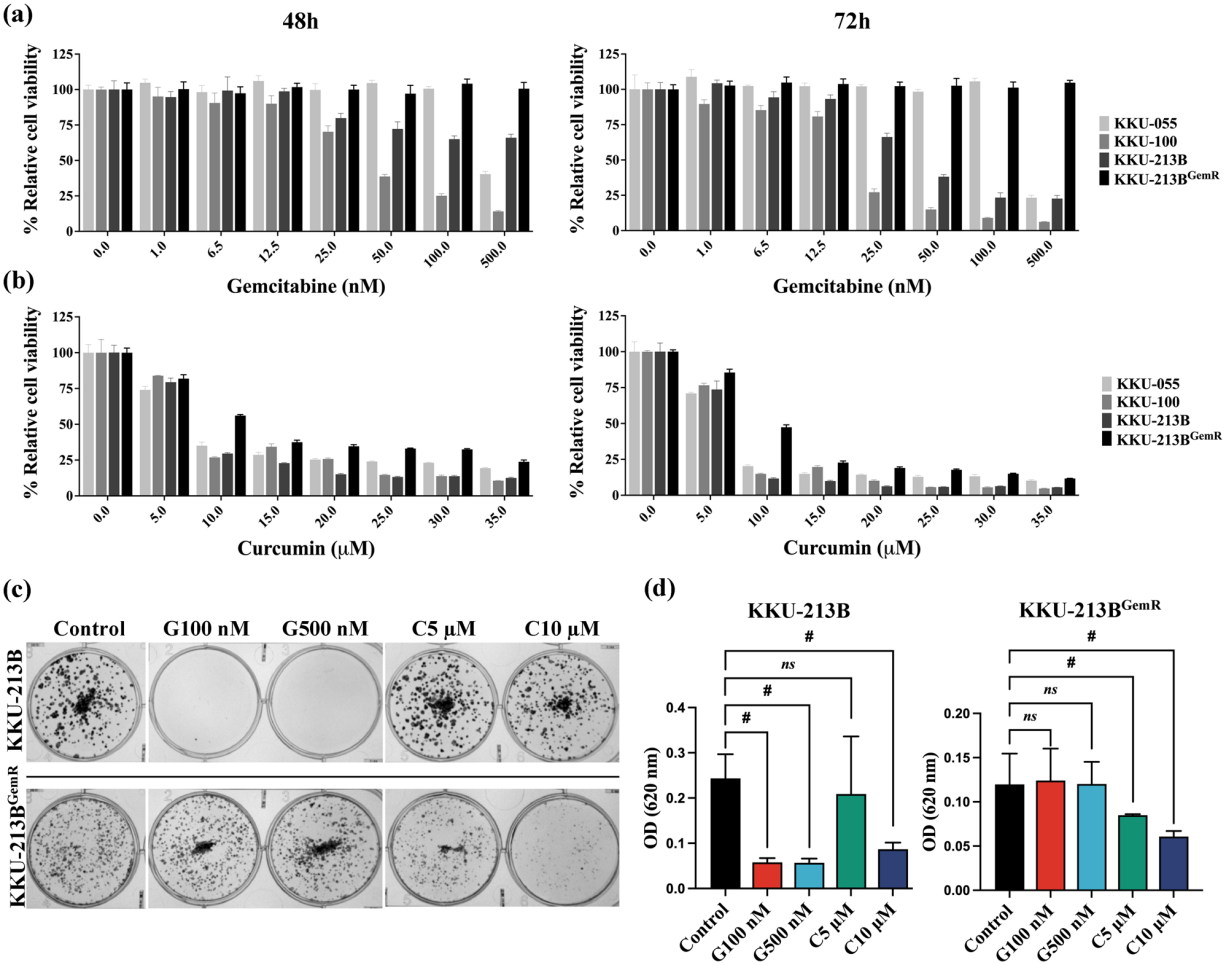
Gemcitabine or curcumin treatment for 48 h and 72 h inhibited cell proliferation (MTT assay) in four CCA cell lines. (**a**) Gemcitabine; 0–500 nM, (**b**) curcumin; 0–35 µM, cell proliferation was calculated based on the untreated control of each cell line and expressed as percentage of relative cell viability. (**c** + **d**) Representative colony formation assays of CCA cell lines treated with gemcitabine or curcumin. All data are expressed as means ± SD of three independent biological replicate experiments; *ns* = not significant, ^#^*p* < 0.0001. G: gemcitabine, C: curcumin.

Anti-CCA activity focusing on cell proliferation was assessed using the MTT assay and clonogenic assay. Curcumin or gemcitabine, administered alone, decreased proliferation of CCA cells (KKU-055, KKU-100, KKU-213B cell lines) in a dose- and time-dependent manner. Interestingly, curcumin also inhibited proliferation of KKU-213B^GemR^ cells in a dose- and time-dependent manner, whereas gemcitabine, as expected, had a limited effect on this cell line (Fig. 2a,b). To further validate these results, we examined their correlation with colony formation results. In KKU-213B cells, gemcitabine and curcumin effectively suppressed colony formation when administered alone, suggesting that they may inhibit CCA cell growth and expansion. Interestingly, in the gemcitabine-resistant KKU-213B^GemR^ cell line, only curcumin showed the ability to inhibit colony formation (Fig. 2c,d), supporting the successful establishment of KKU-213B^GemR^.

### Synergistic effect of curcumin plus gemcitabine against cell proliferation in the gemcitabine-resistant CCA cell line

**Figure 3:**
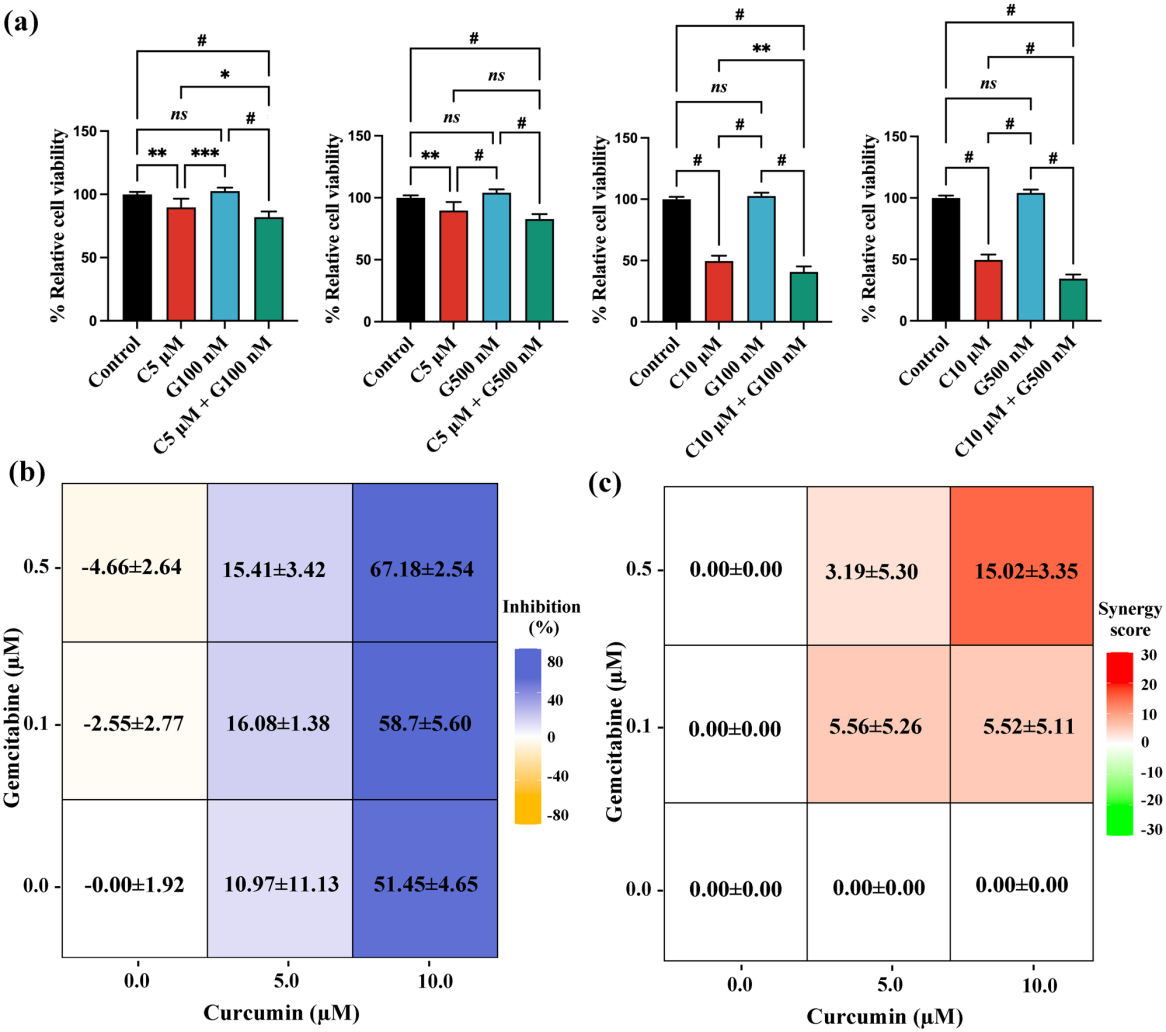
The inhibition of proliferation (MTT assay) by KKU-213B^GemR^ cells after 72 h of incubation with a combination of gemcitabine and curcumin. (**a**) The combination of gemcitabine (100 nM and 500 nM) and curcumin (5 µM and 10 µM). (**b**) Percent inhibition and (**c**) synergy score using the HSA model. All data are expressed as the mean ± SD of biological and technical replicate experiments; *ns* = not significant, **p < 0.01, ***p < 0.001, and ^#^*p* < 0.0001 compared between treatment groups. G: gemcitabine, C: curcumin.

Combination treatment of 5 µM or 10 µM curcumin with either 100 nM or 500 nM gemcitabine enhanced anti-CCA activity against KKU-213B^GemR^ cells (Fig. 3a). Three out of four combinations suppressed cell proliferation significantly more than curcumin or gemcitabine alone. To quantify the synergistic effects of these combinations, we used the highest single agent (HSA) synergy score, where values above 10 indicate synergy, values between − 10 and 10 indicate additive effects, and values below − 10 indicate antagonistic effects. Remarkably, the combination treatments produced a synergistic effect on KKU-213B^GemR^ cells, as evidenced by the highest HSA synergy score of 15.02 ± 3.35 for 67.18 ± 2.54 inhibition (%) using 10 µM curcumin and 500 nM gemcitabine. This was followed by the additive score of 5.52 ± 5.11 and 58.7 ± 5.60 inhibition (%) using 10 µM curcumin and 100 nM gemcitabine. The combination of 5 µM curcumin with either 100 or 500 nM gemcitabine showed additive effects against KKU-213B^GemR^ cells, with synergy scores of 3.19 ± 5.30 and 5.56 ± 5.26, respectively (Fig. 3b,c). This result highlights a cooperative and enhanced antiproliferative effect when curcumin and gemcitabine are used in tandem, especially in gemcitabine-resistant CCA cells.

### Combination of curcumin and gemcitabine synergistically induces cell apoptosis and G2/M phase arrest in gemcitabine-resistant CCA cells (KKU-213B^GemR^)

**Figure 4:**
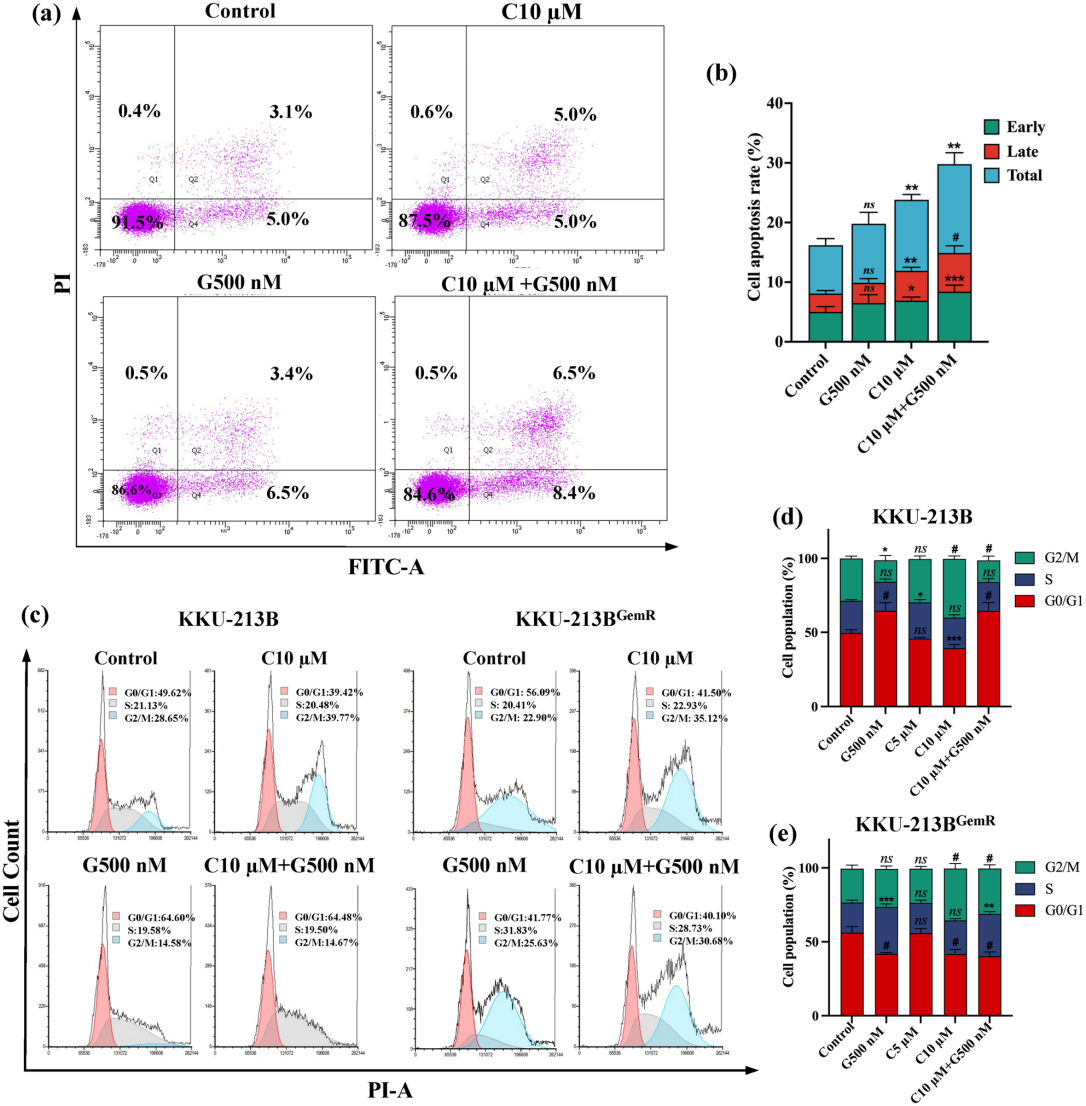
The effects of gemcitabine and curcumin on cell apoptosis (**a**, **b**) and cell cycle arrest (**c**–**e**). (**a**) Flow cytometry analysis using Annexin V-FITC/PI staining, and (**b**) the apoptosis rate (%) of KKU-213B^GemR^ cells under gemcitabine and curcumin treatment. (**c**) KKU-213B and KKU-213B^GemR^ treated with 500 nM gemcitabine and with 5, 10 µM curcumin for 24 h. (**d** + **e**) Percentages of cell populations (%) at each phrase (G0/G1, S and G2M) for KKU-213B and KKU-213B^GemR^. All data are expressed as means ± SD of biological and technical replicate experiments; *ns* = not significant, *p < 0.05, **p < 0.01, ***p < 0.001, and ^#^*p* < 0.0001 compared to control groups. G: gemcitabine, C: curcumin.

The apoptosis analysis of KKU-213B^GemR^ cells revealed significant increases in both early and late apoptosis across treatment groups (curcumin and the combination of curcumin and gemcitabine) compared to the untreated control (Fig. 4a,b). Specifically, curcumin (10 µM) significantly increased early (5.0%,* p* < 0.05) and late (5.0%, *p* < 0.01) stages of apoptosis, resulting in an overall apoptosis rate of 10.0% (*p* < 0.001). The combination treatment significantly boosted early (8.4%, *p* < 0.001) and late (6.5%, *p* < 0.0001) stages of apoptosis, culminating in an overall apoptosis rate of 14.9%. Interestingly, treatment with gemcitabine alone (500 nM) did not produce a significant difference from the untreated controls in both early (3.4%) and late (6.5%) stages of apoptosis, with an overall apoptosis rate of 9.9%. These results indicate that curcumin and the combination treatment produced a significant induction of apoptosis (compared to controls), with the combination of curcumin and gemcitabine exhibiting a synergistic effect, leading to the highest levels of both early and late apoptosis.

Parental CCA cells (KKU-213B) and gemcitabine-resistant CCA cells (KKU-213B^GemR^) were treated with a single dose of curcumin, gemcitabine, or the combination of curcumin and gemcitabine. The percentages of cells in the S and G2/M phases were significantly higher in gemcitabine-treated KKU-213B^GemR^ (31.83 ± 2.08% and 25.63 ± 2.06%, respectively) compared with parent cells KKU-213B (19.58 ± 1.83% and 14.58 ± 3.17%, respectively) (*p* < 0.0001). Treatment of KKU-213B^GemR^ and KKU-213B cells with 10 µM curcumin induced G2/M phase arrest (35.12 ± 3.37% and 39.77 ± 1.97%, respectively). The combination of curcumin and gemcitabine induced arrest in the S and G2/M phases significantly more in KKU-213B^GemR^ (28.73 ± 1.66% and 30.68 ± 2.56%, respectively) than in the parental cells, KKU-213B (19.50 ± 2.28% and 14.67 ± 2.85%) (*p* < 0.0001) (Fig. 4c–e). These results indicate that the gemcitabine-resistant and parental CCA cells respond differently to the combination of these two drugs, suggesting a shift in cell cycle regulation patterns.

### Overexpression of LAT2 protein in gemcitabine-resistant CCA cells

**Figure 5:**
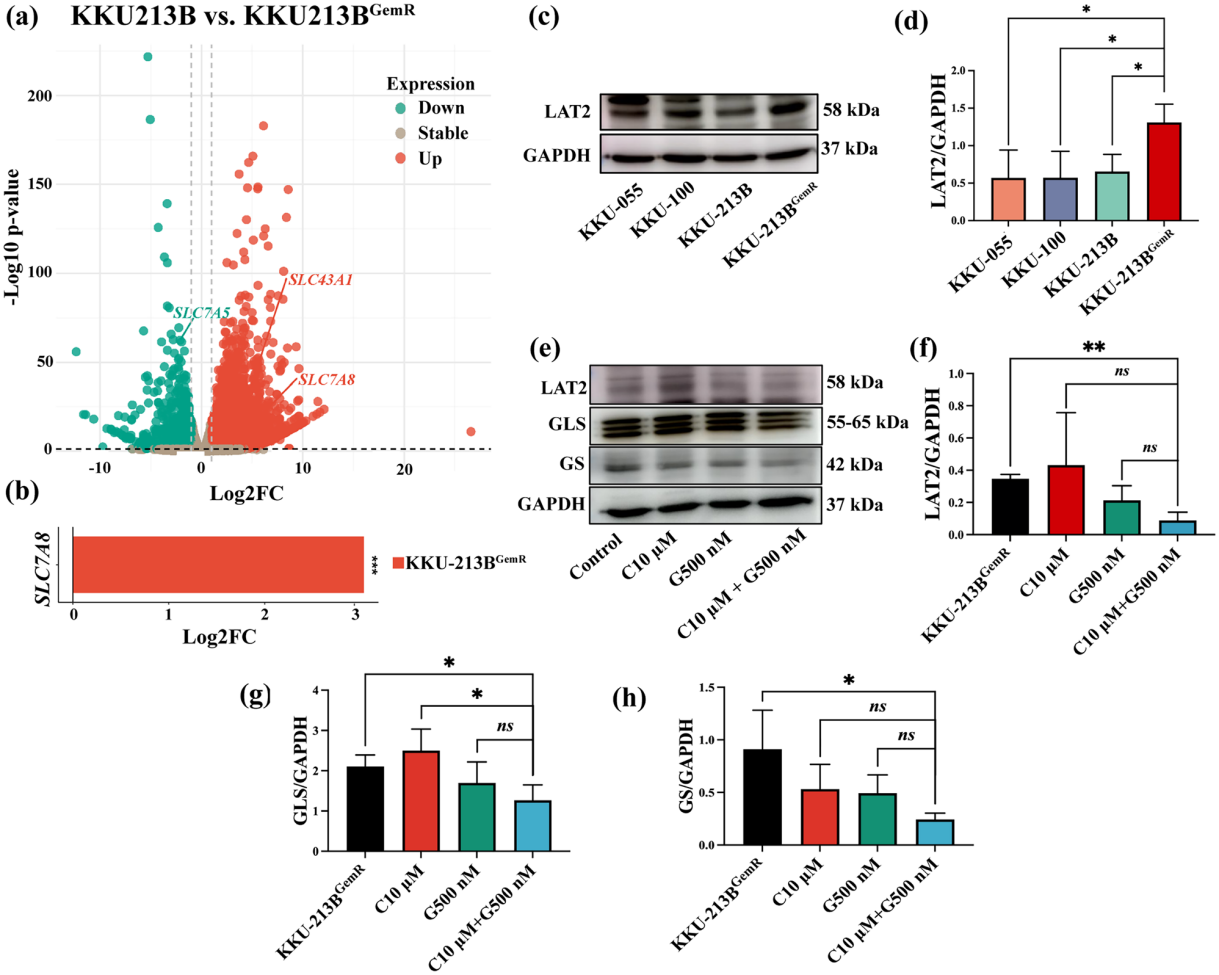
Overexpression of LAT2 protein in gemcitabine-resistant CCA cells. (**a**) Volcano plot of differentially expressed genes of KKU-213B (parental) vs. KKU-213B^GemR^. Green dots indicate downregulated genes, orange dots indicate upregulated genes, and brown dots indicate stable genes. (**b**) The fold change of *SLC7A8* expression in KKU-213B compared to KKU-213B^GemR^. (**c** + **d**) LAT2 expression and intensity in parental CCA cells and gemcitabine-resistant CCA cells and (**e**–**h**) LAT2, glutaminase (GLS) and glutamine synthetase (GS) expression and intensity after treatment with gemcitabine, curcumin and the combination of curcumin and gemcitabine. Original blots are presented in Supplementary Table S2. Data are expressed as mean ± SD of three replicate experiments; *ns* = not significant, *p < 0.01, **p < 0.01 and ***p < 0.001 compared between groups; Log2FC: Log2 fold-change.

We identified over 50,000 genes, encompassing both non-coding and coding genes. The volcano plot (Fig. 5a) illustrates the up-regulation and down-regulation of genes, with a specific focus on genes associated with the solute-carrier family, such as *SLC7A5, SLC43A1*, and *SLC7A8.* Our findings revealed that *SLC43A1* and *SLC7A8* were up-regulated in KKU-213B^GemR^ compared to KKU-213B (*p* < 0.05) (Fig. 5a). Notably, *SLC7A8*, a gene linked to chemoresistance, exhibited a three-fold increase in expression in KKU-213B^GemR^ compared to KKU-213B (*p* < 0.001) (Fig. 5b). To establish a more compelling link between *SLC7A8* gene and the development of gemcitabine resistance in CCA cells. We compared LAT2 protein (encoded by *SLC7A8*) expression across three CCA cell lines (KKU-055, KKU-100, and KKU-213B) and one gemcitabine-resistant CCA cell line (KKU-213B^GemR^). Interestingly, LAT2 was found to be overexpressed in KKU-213B^GemR^ compared with the other cell lines (Fig. 5c,d). In addition, we determined the effect of the combination of curcumin and gemcitabine on the expression of LAT2, glutaminase (GLS), and glutamine synthetase (GS) proteins. This demonstrated that KKU-213B^GemR^ cells treated with this combination showed a significant suppression in the intensity of LAT2 (*p* < 0.01), GLS (*p* < 0.05), and GS (*p* < 0.05) protein compared to the untreated controls (Fig. 5e–h).

**Figure 6:**
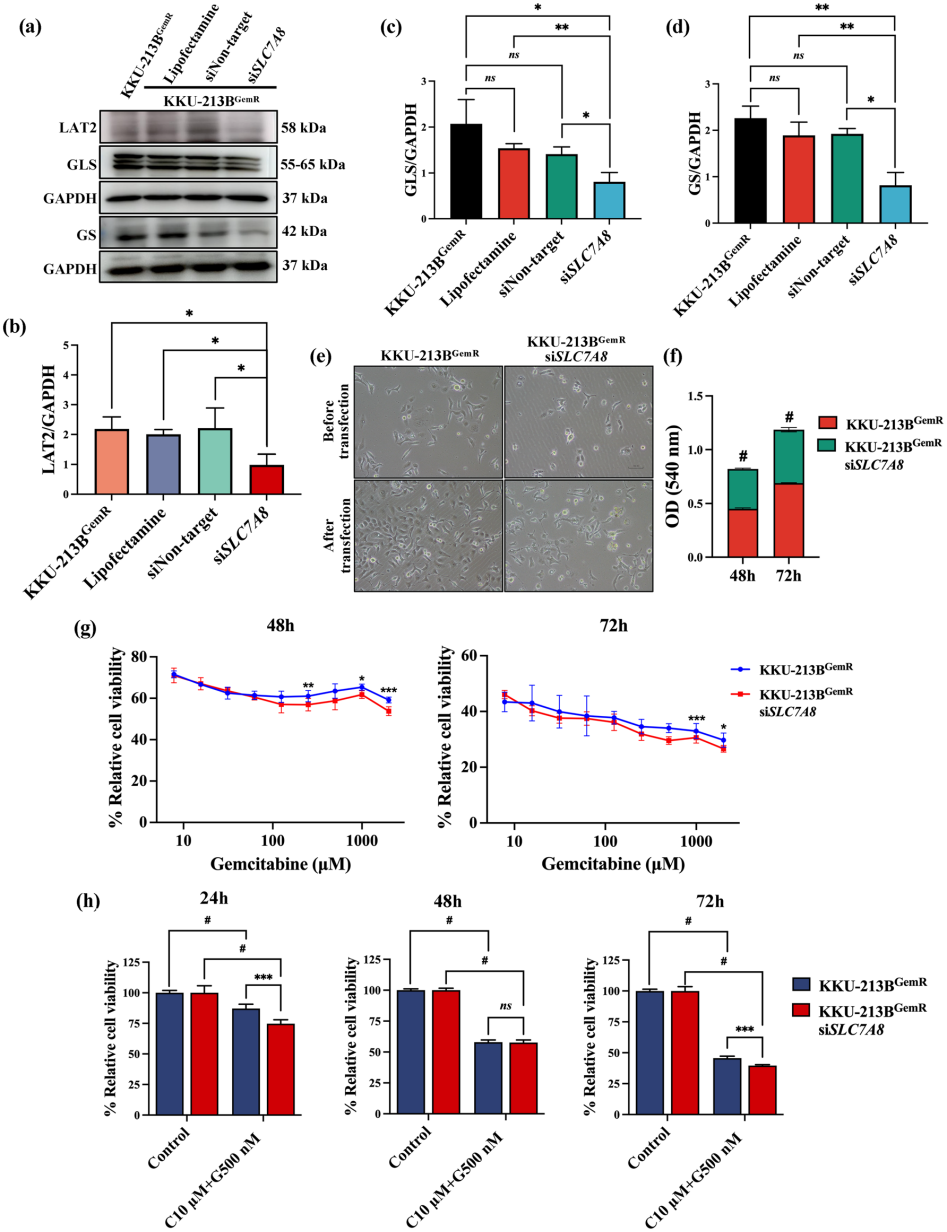
LAT2 promotes chemotherapeutic sensitivity against gemcitabine-resistant cell. Western blot analyses of (**a**–**d**) LAT2, glutaminase (GLS) and glutamine synthetase (GS) in KKU-213B^GemR^ knockdown *SLC7A8*. (**e**) Cell morphology and confluence of cells before and after transfection at 48h (10X magnification). (**f**) Cell proliferation using MTT assay after transfection with si*SLC7A8* at 48 h and 72 h (OD 540), treatment with (**g**) gemcitabine 0–2000 μM at 48 h and 72 h and (**h**) the combination of curcumin and gemcitabine at 24–72 h. Original blots are presented in Supplementary Table S3. Western blot data are expressed as mean ± SD of biological tripicates and cell proliferation are expressed as mean ± SD of biological and technical replicate experiments; *ns* = not significant, *p < 0.05, **p < 0.01, ***p < 0.001 and ^#^*p* < 0.0001 compared to control and between groups. G: gemcitabine, C: curcumin.

We then performed siRNA-mediated *SLC7A8* knockdown experiments in KKU-213B^GemR^ and obtained an impressive knockdown efficiency of 55.62%, with a significant decrease in the expression of LAT2 in the transfected cells compared with non-transfected KKU-213B^GemR^ (Fig. 6a,b). Moreover, our results demonstrated a significant decrease in expression level of GLS protein in KKU-213B^GemR^ si*SLC7A8* cells compared to the controls, comprising non-transfected cells (*p < *0.05), cells treated with Lipofectamine (*p* < 0.01) and siNon-target gene (*p* < 0.05) (Fig. 6c). Additionally, GS exhibited a significant decrease compared to non-transfected cells (*p* < 0.01), cells treated with Lipofectamine alone (*p* < 0.01) and siNon-target gene (*p *< 0.05) (Fig. 6d). KKU-213B^GemR^ si*SLC7A8* exhibited other noticeable effects, particularly slower cell growth compared to KKU-213B^GemR^ non-transfected cells. The proliferation of KKU-213B^GemR^ si*SLC7A8* cells was significantly lower than non-transfected cells at 48–72 h post-transfection (*p* < 0.0001) (Fig. 6e,f). Moreover, MTT assays demonstrated that KKU-213B^GemR^ si*SLC7A8* exhibited significantly greater sensitivity to gemcitabine than KKU-213B^GemR^ non-transfected cells, indicated by lower cell proliferation. At 48 h, significant differences were observed at doses of 250 μM (*p* < 0.01), 1000 μM (*p* < 0.05), 2000 μM (*p* < 0.001). Similary, at 72 h, significant differences were noted at doses of 1000 μM (*p* < 0.001), 2000 μM (*p* < 0.05) (Fig. 6g). Interestingly, KKU213^GemR^ si*SLC7A8* treated with the combination exhibited a significantly greater reduction in cell proliferation compared to KKU-213B^GemR^ non-transfected cells treated with the same combination at both 24 h and 72 h intervals (*p* < 0.001) (Fig. 6h).

These significant results demonstrate the central role of *SLC7A8* in gemcitabine resistance of CCA. The observed reduction in cell proliferation and increased sensitivity to gemcitabine after *SLC7A8* knockdown in KKU-213B^GemR^ cells, along with the potential reduction in LAT2 when treated with a combination of curcumin and gemcitabine, likely lead to decreased metabolism of glutamine pathway and strongly suggest a direct link between LAT2 and gemcitabine-resistance in cholangiocarcinoma. This insight into the involvement of LAT2 opens new avenues for understanding and potentially overcoming gemcitabine-resistance in CCA. Employing *SLC7A8* targeting, along with a combined curcumin and gemcitabine treatment, may be novel approach to treatment of gemcitabine-resistant CCA.

### Combination of curcumin and gemcitabine suppresses tumor progression and decreases the level of LAT2, CK19 and HMGB1 in a gemcitabine-resistant CCA xenograft mouse model and in a hamster CCA model

**Figure 7:**
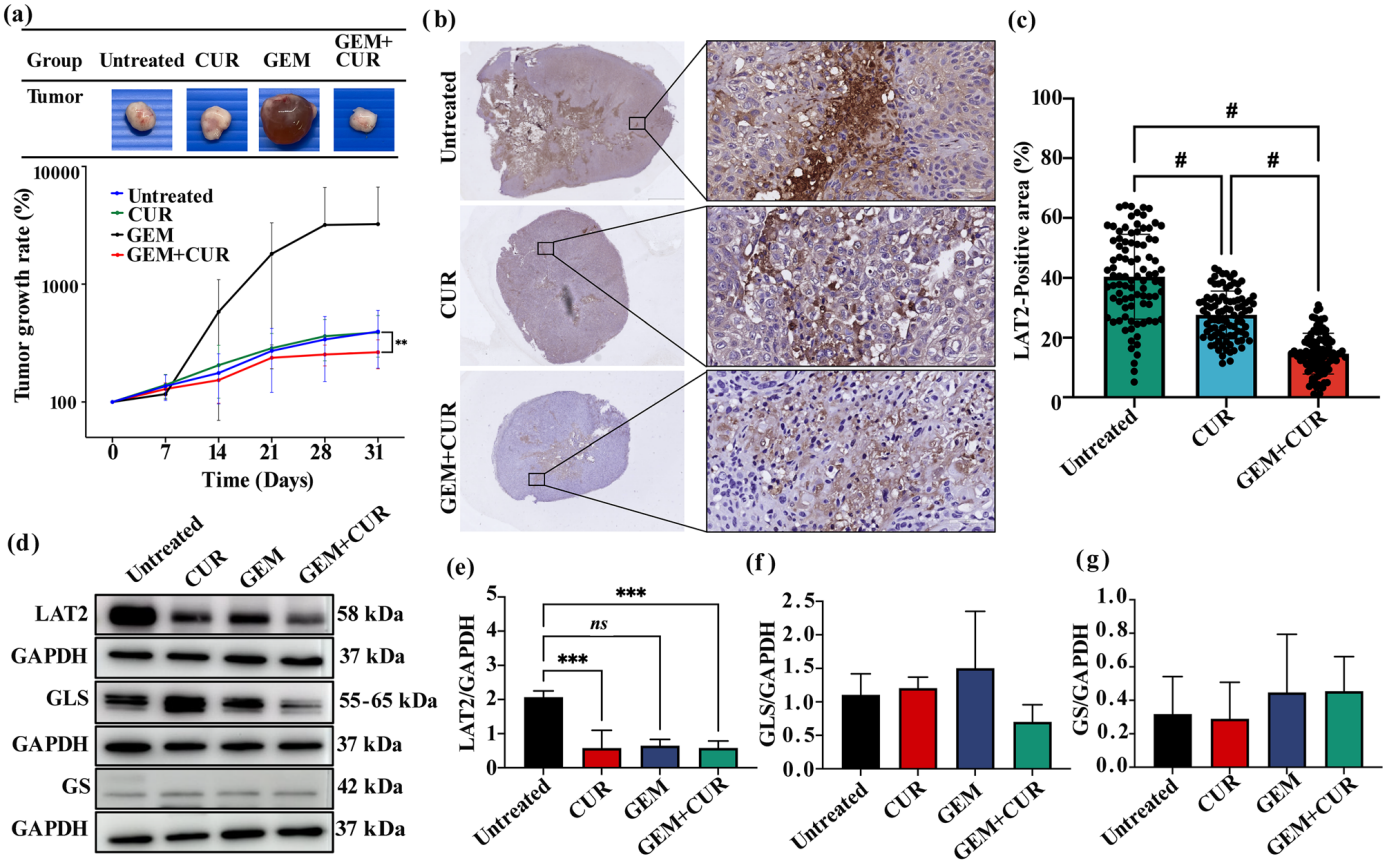
Anti-tumor effects of curcumin, gemcitabine and their combination in gemcitabine- resistant xenograft mouse model. (**a**) Tumor growth rates and images of tumor tissue (N = 5 in each group), (**b**) LAT2 immunohistochemical staining in the untreated group, curcumin- and combination-treated groups (the gemcitabine-treated group was not included due to fluid accumulation in the tumor mass) and (**c**) the positive-staining area (%) of LAT2. (**d**–**g**) Protein intensities of LAT2, GLS and GS. Original blots are presented in Supplementary Table S4. All data are expressed as means ± SD of three biological independent experiments; *ns* = not significant, ** *p* < 0.01, ***p < 0.001 and ^#^*p* < 0.0001 compared between groups (N = 3 in each group). CUR: Curcumin, GEM: Gemcitabine.

We tested the efficacy of a combination of curcumin and gemcitabine in a xenograft tumor model using KKU-213B^GemR^ cells. Treatment with either gemcitabine or curcumin alone did not result in a significant difference in tumor growth rate compared to the control group. Specifically, the gemcitabine treated group exhibited the highest tumor growth rate at the endpoint, with a rate of 3238%, whereas the curcumin treated group and the control group showed growth rates of 464% and 391%, respectively. Notably, the group receiving the combined treatment demonstrated the lowest tumor growth rate of 265%, significantly lower than seen in the control group (*p* < 0.01) (Fig. 7a). Immunohistochemical analysis revealed a significant decrease in the LAT2 positive area in groups treated with curcumin alone and in combination with gemcitabine compared to the untreated group (*p* < 0.0001) (Fig. 7b,c). Notably, in the gemcitabine-treated group, there was fluid accumulation in the tumor mass, preventing its use for immunohistochemical study. Nevertheless, the combination treatment group exhibited a significantly greater reduction in LAT2 positive area compared to the curcumin treated group, indicating a synergistic effect (*p* < 0.0001) (Fig. 7c). At the protein level (Fig. 7d–g), the combination of curcumin and gemcitabine significantly suppressed LAT2 expression (*p* < 0.001) (Fig. 7d,e). However, the intensity of GLS and GS did not show significant differences in any treatment group compared to the untreated group (Fig. 7d,f,g).

In addition, we successfully induced the development of CCA in hamsters, using *O. viverrini* infection and administration of NDMA, to confirm the efficacy of the combination of curcumin and gemcitabine found in xenograft mouse model. Our results showed remarkable differences in nodule formation between the experimental groups. In the OV + NDMA + Untreated group, multiple white nodules (size 0.3–0.5 cm) were observed. In the curcumin- and gemcitabine-treated groups, 1–2 white nodules (size 0.3–0.4 cm and 0.2–0.8 cm, respectively) were found. In contrast, the group treated with the combination of curcumin and gemcitabine displayed only small nodules (size less than 0.3 cm) in the livers (Supplementary Fig. S2a). Hematoxylin and eosin staining provided supporting evidence (Supplementary Fig. S2b,c). In the OV + NDMA + Untreated group, the lesions were classed as cholangiocarcinoma (4+, 44.44% of lesions), cholangiofibrosis (2+, 22.22%), chronic inflammatory cells (2+, 22.22%) and cholangiofibroma (1+, 11.11%). In the OV + NDMA + CUR group, the lesions were cholangiofibrosis (2.5+, 38.46%), followed by cholangiocarcinoma (2+, 30.77%), chronic inflammatory cells (1+, 15.38%) and cholangiofibroma (1+, 15.38%). In the OV + NDMA + GEM group the lesions were classed as chronic inflammatory cells (3.5+, 36.48%) following by cholangiofibrosis (3+, 31.58%), cholangiocarcinoma (2+, 21.05%), and cholangiofibroma (1+, 10.53%). Moreover, this group exhibited cholangitis and fluid accumulation correlated with in the xenograft model. The OV + NDMA + GEM + CUR group did not show lesions of cholangiocarcinoma and cholangiofibroma; only cholangiofibrosis (2+, 66.67%) and chronic inflammatory cells (1+, 33.33%) were found. Supportively, the extent of CK19- and HMGB1-positive areas in the CCA control group were significantly higher than in all treatment groups (*p* < 0.0001). CK19 and HMGB1-positive areas did not differ significantly between the gemcitabine-treated and curcumin-treated groups. Interestingly, the combination-treated group showed significantly lower CK19 and HMGB1 levels than the curcumin-treated group (*p* < 0.01 and *p* < 0.001, respectively) and the gemcitabine-treated group (*p* < 0.001 and *p* < 0.01, respectively) (Supplementary Fig. S2d,e). Overall, while neither gemcitabine or curcumin treatment alone significantly affected tumor growth, a combination of the two markedly inhibited tumor growth and attenuated the histopathologic features associated with CCA progression in both hamster and xenograft mouse models. This suggests a potential synergistic effect of curcumin and gemcitabine.

## Discussion

**Figure 8:**
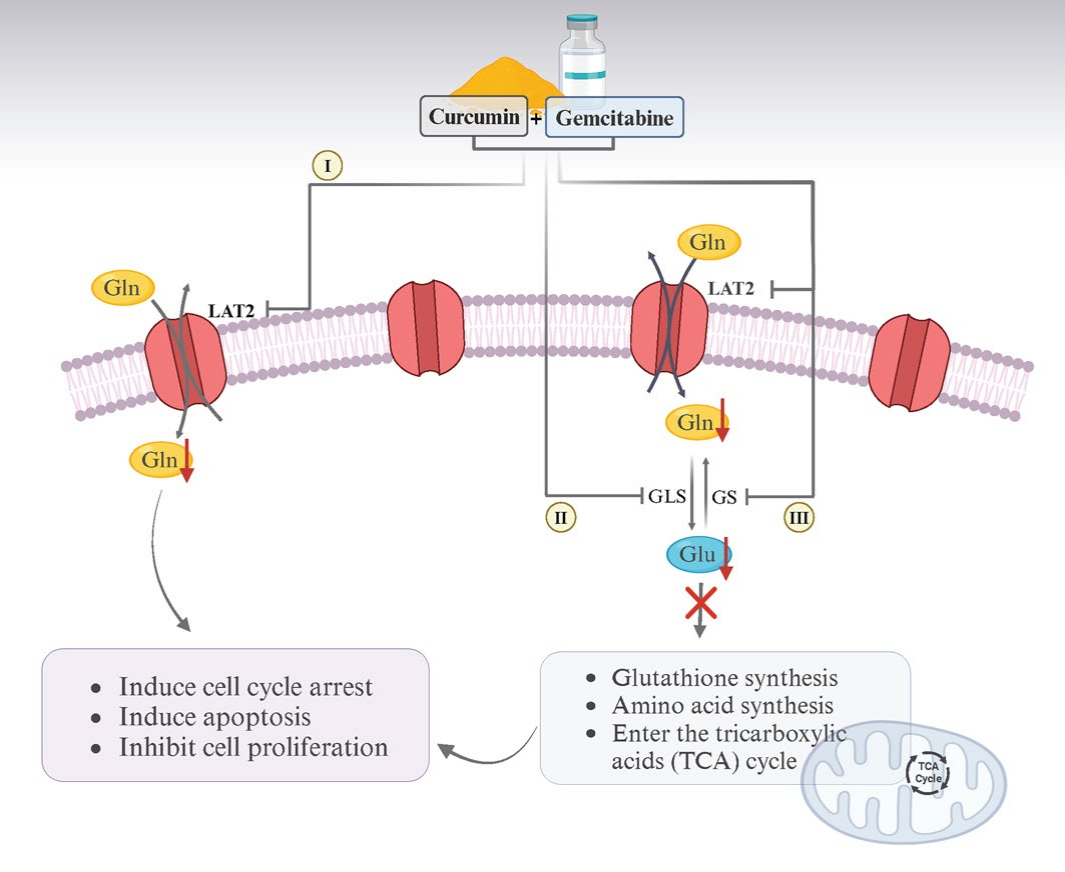
This summary schematic demonstrates that curcumin enhances gemcitabine's ability to (I) suppress the *SLC7A8* (LAT2)-regulated glutamine pathway, (II) inhibit GLS, and (III) inhibit GS. This leads to reduced ability of glutamine (Gln) to participate in synthesis of nucleotides, and of glutamate (Glu) being unable to enter the tricarboxylic acid (TCA) cycle and synthesize glutathione and amino acids^19^. These mechanisms result in inhibited cell proliferation, induced cell cycle arrest, and apoptosis, ultimately inhibiting cancer progression in gemcitabine-resistant cholangiocarcinoma.

Cholangiocarcinoma is a cancer with poor prognosis. Patients usually present at a late stage, leading to high morbidity and mortality, and poor response to drug treatment. Here, we established a drug-resistant CCA cell line (KKU-213B^GemR^) to test the therapeutic effect of a combination of curcumin and gemcitabine in CCA both in vivo and in vitro, especially in resistant CCA cell lines. The results showed that the combination of curcumin and gemcitabine was effective in treating resistant CCA cells, in part reducing expression of the LAT2/glutamine pathway, as summarized in Fig. 8.

Previously, gemcitabine-resistant CCA cell lines (using as parents KKU-M139 and KKU-M214) were established using an approach similar to ours. The resultant KKU-M139/GEM and KKU-M214/GEM cells were 25.88- and 62.31-fold more resistant to gemcitabine than their parent cells with low expression of the cell cycle distribution protein that leads to G2/M arrest^20^. Using RBE-R and QBC939-R cells, other researchers have successfully constructed gemcitabine-resistant CCA cell lines after 8 months of induction. They found an increase of IC_50_ values from 3.011 µM to 14.44 µM for RBE-R and 4.501 to 18.19 µM for QBC939-R. Cell cycle arrest tended to occur at the S phase in resistant cells^21^. Our gemcitabine-resistant CCA cell line showed 5.50- and 26.22-fold higher resistance to gemcitabine than the parental cells at 40 and 60 cycles after gemcitabine induction, respectively. This could be related to the different doses of gemcitabine treatment (Figs. 1b and 6e). These results suggest that prolonged and repeated exposure of KKU-213B cells to gemcitabine induces greater resistance to the drug in this cell line.

Curcumin inhibits the activation of oncogenic transcription factors, decreasing the level of anti-apoptotic proteins, and promoting the expression of pro-apoptotic proteins^9,10^. Moreover, curcumin enhances the efficacy of gemcitabine treatment in pancreatic cancer by inducing apoptosis of pancreatic cancer cells, downregulating NF-κB-regulated gene products, suppressing angiogenesis and cell migration^22,23^. Our results showed that curcumin suppresses cell proliferation and colony formation of CCA cells by inducing apoptosis and arresting the cell cycle. The combination of curcumin and gemcitabine significantly enhanced apoptosis compared to either treatment alone. Furthermore, curcumin-treated cells exhibited a tendency to accumulate at the G2/M stage in both parental and gemcitabine-resistant CCA cells. Interestingly, the synergistic effect of curcumin and gemcitabine induced accumulation in the S and G2/M phases in our gemcitabine-resistant CCA cells more effectively than either curcumin or gemcitabine treatment alone. The results are consistent with previous findings regarding the effects of curcumin in various types of cancer^12,24–26^.

Amino acid transporters play an essential role in amino-acid transport, tumor progression, and therapy resistance^27^. *SLC7A8* is a member of solute carrier family 7 (SLC7), which encodes the LAT2 protein involved in chemoresistance in various cancers^18,28,29^. LAT2 is a transmembrane protein for transportation of neutral amino acids such as glutamine. Our results indicate that *SLC7A8* is overexpressed in gemcitabine-resistant CCA cells compared to parental cells. Knockdown with si*SLC7A8* had more than 55% efficacy. Furthermore, the results of increased gemcitabine sensitivity and decreased cell growth after knockdown by si*SLC7A8* support the involvement of the *SLC7A8* in chemoresistance in cholangiocarcinoma. Moreover, LAT2 was suppressed by this combination of curcumin and gemcitabine both in gemcitabine-resistant CCA cell lines and the xenograft mouse model. Supportively, Feng et al. found that overexpression of LAT2 in a pancreatic xenograft mouse model led to reduced sensitivity to gemcitabine, as indicated by a non-significant difference in tumor volume between the gemcitabine-treated group and the control group^18^. Conversely, overexpression of *SLC7A8* has been shown to inhibit growth and migration in lung adenocarcinoma^30^ and is associated with good prognosis in estrogen receptor-positive breast cancer^17^. Interestingly, according to data from the Cancer Genome Atlas (TCGA), solute carrier (SLC) transporters can have either favorable or unfavorable prognostic value for the different tumor entities^31^. The divergent prognostic impacts may stem from differences in tumor microenvironment, metabolic dependencies, and the genetic or epigenetic context of the cancer. This environment can vary greatly between different types of cancer and even between patients with the same type of cancer^32,33^.

Down-regulation of LAT2 may reduce glutamine uptake of the cancer cells. Glutamine is metabolized by glutaminase (GLS) and glutamine synthetase (GS or GLUL) to glutamate that is involved in amino acid glycolysis, amino acid metabolism, nucleotide metabolism, fatty acid metabolism and the tricarboxylic acid (TCA) cycle, processes associated with tumor progression and the modulation of the tumor microenvironment (TME). Elevated expression of GLS1 correlates with poor prognosis in various cancer types, including CCA, by inducing metastasis and invasion, and promoting the epithelial-mesenchymal transition^34,35^. Similarly, the overexpression of GS, is associated with poor prognosis in various cancer types such as breast cancer^36^, glioblastoma^37^, prostate cancer^38^ and hepatocellular carcinoma^39^. Therefore, targeting GLS and GS presents a promising therapeutic strategy. In our result, we found that the expression of GLS and GS decreased significantly after knockdown of KKU-213B^GemR^ with si*SLC7A8* and treatment with a combination of curcumin and gemcitabine. This is consistent with previous findings where curcumol and the combination of curcumin and cisplatin attenuated glutaminase activity in hepatic stellate cells and colon cancer cells^14,39,40^. Conversely, in pancreatic cancer cells, GLS levels increased and GS levels decreased after *SLC7A8* knockdown^18^. These somewhat contradictory findings may be due to variations in the complex nature of carcinogenesis, the TME and the metabolic demands of each cancer type^19,41,42^. Moreover, our gemcitabine-resistant CCA model was consistent with in vitro results by suppressing tumor growth rate and LAT2 staining intensity. Although GLS did not show significant differences compared to the untreated group, the combination treatment group showed a trend toward decreased GLS. The efficacy of the combination of curcumin and gemcitabine in a gemcitabine-resistant cancer xenograft model was supported in a previous study^12^.

In order to mimic human *Opisthorchis viverrini*-associated cholangiocarcinoma (CCA), we evaluated the synergistic effects of a combination of curcumin and gemcitabine in an orthotopic hamster CCA model. This model typically originates from infection-induced inflammation and chronic irritation, as documented in previous studies^43,44^. Our results showed that the combination group displayed mainly cholangiofibrosis, a precancerous lesion, without the appearance of tumor foci. In contrast, although cancerous lesions trended to fewer in both the curcumin and gemcitabine groups than in the untreated control group, CCA was still present. The combination group also decreased the intensity of cytokeratin 19 (CK19) and high-mobility group box 1 (HMGB1) staining in liver tissues, markers for confirming the bile-duct epithelial origin of CCA and inflammation-associated carcinogenesis, respectively^45–47^, leading to the inhibition of CCA progression in the hamster model. This finding consistent with our previous results^11,48,49^. A preliminary study in hamster model strongly supports findings in a xenograft model. To achieve a better understanding of the underlying mechanism, larger sample sizes in the hamster CCA model and *SLC7A8* knockout in a gemcitabine-resistant xenograft model are required for future studies.

In conclusion, our study shows that the combination of curcumin and gemcitabine synergistically exert an anticancer effect, especially in gemcitabine-resistant CCA cells. Moreover, our in vivo data confirm the efficacy of this combination therapy. Specifically, we identified that *SLC7A8* (LAT2) regulates the glutamine mechanism pathway involved in cancer progression. This pathway is a compelling candidate for association with chemotherapy resistance, and our results demonstrate a clear link between LAT2 protein expression and gemcitabine resistance both in vitro and in vivo. These results highlight the potential of the synergistic effects of curcumin and gemcitabine, together with targeting the LAT2*/*glutamine pathway, as a promising strategy for the treatment of CCA and possibly other cancers.

## Materials and methods

### Cell lines

This study was approved by Khon Kaen University Human Research Ethics Committee based on the Declaration of Helsinki and Good Clinical Practice Guidelines ICH (HE651479). Informed consent was written from each patient as previously established by Dr. Banchob Spira (Khon Kaen University)^50,51^. Four human CCA cell lines representing different phenotypes of CCA were used: KKU-055, KKU-100, KKU-213B and KKU-213B^GemR^. The gemcitabine-resistant CCA cell line (KKU-213B^GemR^) was newly established from KKU-213B. All cell lines were cultured in Dulbecco’s modified Eagle’s medium (DMEM)(Gibco, Grand Island, NY, USA) supplemented with 10% fetal bovine serum (FBS) (Corning, NY, USA), 100 U/ml penicillin and 100 µg/ml streptomycin (Gibco). All cells were grown at 37 °C and 5% CO_2_ in a humidified incubator.

### Establishment and characterization of drug-resistant (KKU-213B^GemR^)

The gemcitabine-resistant CCA cell line, KKU-213B^GemR^, was established by growing the parental KKU-213B cell line in DMEM supplemented with 10% FBS, 100 U/ml penicillin, 100 µg/ml streptomycin, and 5 µM gemcitabine (GEMITA, Fresenius Kabi, India). Cells were maintained under these conditions for 72 h in a humidified incubator. Then the culture medium was replaced with fresh culture medium. After the cells reached 90% confluence, they were sub-passaged and cultured in the presence of 5 µM gemcitabine. After repeating this for 40 cycles, gemcitabine resistance was assessed using the MTT assay (Invitrogen, Thermo Fisher Scientific, MA, USA). In all experiments, gemcitabine induction included up to 60 cycles.

### Library preparation and RNA sequencing

Total RNA was extracted from three biological replicates each of KKU-213B and KKU-213B^GemR^ using RNA extraction kit (Invitrogen) following the manufacturer’s instruction. The RNA samples were evaluated for degradation and contamination using a 1% agarose gel. Purity of the RNA was determined with the NanoPhotometer^®^ spectrophotometer (IMPLEN, CA, USA), and RNA integrity and quantity were measured using the RNA Nano 6000 Assay Kit on the Bioanalyzer 2100 system (Agilent Technologies, CA, USA). Whole-transcriptome sequencing was performed using paired-end sequencing (2 × 150 bp) on a NovaSeq high-throughput sequencer (Illumina, Inc., San Diego, CA, USA). HISAT2 alignment program (cite: https://www.nature.com/articles/nmeth.3317) was used for mapping RNA-seq reads to a reference human genome. The read counts were normalized, and differential gene expression analysis performed with R language using the DESeq2 package (version: 1.40.2). The cutoff-value for differential expression analysis was log2 fold-change > 1.0 with *p*-value < 0.05. Volcano and bar plots were created with ggplot2 (version: 3.4.4).

### Short tandem repeat (STR) analysis

Genomic DNA was extracted from KKU-213B and KKU-213B^GemR^ using the genome extraction kit (QIAGEN, Valencia, CA, USA). Highly polymorphic STR loci (13 autosomal loci and 18 X-chromosome loci) and the gender marker (amelogenin) were analyzed using the AmplFLSTR identifier PCR Amplification Kit^51^ (Thermo Fisher Scientific, Applied Biosystems, MA, USA).

### Measurement of cell proliferation using the MTT assay

CCA cells were seeded at 2000 cells per well in flat-bottomed 96-well plates (Corning). Starting the following day, cells were incubated for 24–72 h with either various concentrations of curcumin (> 97% purity w/w, Merck-Schuchardt, Hohenbrunn, Germany), gemcitabine, or a combination of curcumin and gemcitabine, in a humidified incubator. The culture medium was removed and replaced by 100 µl DMEM without FBS or antibiotic. 10 µl of MTT reagent was added to a final concentration of 0.5 mg/mL and the plates were incubated in a humidified incubator for 2 h. The culture medium was then removed and 100 µL of DMSO was added to solubilize the cells. The absorbance at 540 nM was determined using the Varioskan™ microplate reader (Thermo Fisher Scientific, MA, USA). The absorbance of untreated cells was used as a control. The synergistic effect was calculated using SynergyFinder Plus^52^. Half-maximal inhibitory concentration (IC_50_) was determined using the dose–response inhibition model available in GraphPad Prism 9.0 (GraphPad Software, Inc., CA, USA).

### Clonogenic assay

KKU-213 and KKU-213^GemR^ cells were seeded at 1000 cells per well in 6-well plates and grown overnight in culture medium. Cells were then incubated for 24 h with either curcumin or gemcitabine. After 24 h, the cells were transferred to normal medium and allowed to undergo colony formation for 7 days (KKU-213B) or 12 days (KKU-213B^GemR^). Then colonies were fixed in 4% paraformaldehyde solution and stained with crystal violet. Finally, the colonies were dissolved with 33% acetic acid and absorbance determined with a microplate reader at 620 nm.

### Apoptosis

KKU-213^GemR^ cells were seeded in 6-well plates 2 × 10^5^ cells/well (Corning) and incubated in culture medium for 24 h. Subsequently, the cells were treated with curcumin, gemcitabine, or a combination of both for 72 h. After treatment, the supernatant and cells were collected, washed with ice-cold 1X PBS, and stained using a dual staining method with Annexin V-FITC and propidium iodide (PI) (BioLegend, San Diego, CA, USA), according to the manufacturer's instructions. Apoptotic cells were quantified using a BD FACSCanto II flow cytometer (BD Biosciences, San Jose, CA, USA), and the data were analyzed with FlowJo™ software version 10.8.1 (Becton, Dickinson & Company, OR, USA).

### Cell cycle arrest

KKU-213 and KKU-213^GemR^ cells were seeded in 6-well plates at a density of 2 × 10^5^ cells/well and grown in culture medium for 24 h. Cells were then treated with either curcumin, gemcitabine, or the combination of curcumin and gemcitabine for 24 h. Cells were collected, washed with ice-cold 1X PBS, and fixed overnight with 70% ethanol at − 80 °C. The fixed cells were spun down and washed with ice-cold 1X PBS. Then, the cells were stained with FxCycle™ PI/RNase solution (Molecular Probes, Life Technologies, CA, USA) according to the manufacturer’s guidelines. Finally, the stained cells were passed through a BD FACSCanto™II flow cytometer and the data were analyzed using BD FACS Diva software (BD Biosciences) and FCS Express 7 (De Novo Software, Los Angeles, CA).

### RNA interference

Human *SLC7A8* siRNA, synthesized by Dharmacon Research (Thermo Fisher Scientific), hits five GenBank accessions; NM_001267036, NM_001267037, NM_012244, NM_182728, and NR_049767. KKU-213^GemR^ was transfected with si*SLC7A8* at 120 pmol packed with Lipofectamine 2000 (Invitrogen) in 6-well plates (7.5 × 10^4^ cells/well). Then, KKU-213^GemR^si*SLC7A8* cells were harvested for the study of cell proliferation and protein expression.

### Western blot analysis

CCA cells and tumor tissue were lysed with 1X RIPA reagent (Cell Signaling Technology, Danvers, MA, USA) and the protein concentration was measured with Pierce BCA Protein Assay Kit (Thermo Fisher Scientific Inc). Total protein lysates (20 ug) were separated using the SDS-PAGE technique and transferred to a PVDF membrane (Cytiva, Dreieich, Germany). Membranes were block with 1 min protein-free blocking buffer (BlockPRO™, Energenesis biomedical co., ltd, Taipei, Taiwan) for 1 h and then incubated overnight at 4 °C with primary antibodies (1:1000) including *SLC7A8* (LAT2) (A14861/A24043), GS (A5437) and GLS (A11043), which were purchased from abclonal in Wuhan, China, and GAPDH (sc-25778), which was purchased from Santa Cruz Biotechnology in Dallas, TX, USA. Membranes were probed with the secondary antibody horseradish peroxidase (HRP)-conjugated goat anti-rabbit IgG (1:2000) (Jackson Immuno Research Inc., West Grove, PA, USA). Proteins were visualized using an enhanced chemiluminescence detection system (EMD Millipore Corporation, MA, USA) and protein staining intensity was quantified using National Institutes of Health (NIH) ImageJ and calculated as a ratio to GAPDH. The originals of the western blot images are provided in the Supplementary Table  2-4.

### Parasites

Pepsin digestion was performed as described elsewhere ^53^. Briefly, *O. viverrini* metacercariae were isolated from naturally infected cyprinid fish by pepsin digestion. Fish were ground in a blender with pepsin-HCl. The digest was placed in a plastic bucket and incubated for 1 h in a shaking water bath at 37 °C. The digested material was filtered through a series of sieves of different pore sizes into a conical sedimentation plastic container containing 0.85% NSS. The digestion solution was allowed to stand for 15–30 min to form a sediment. Metacercariae of *O. viverrini* were isolated from the sediment and counted under a dissecting microscope. Viable cysts (showing movement of the worm within the cyst) were used for hamster infection.

### Experimental animals

All animal experimental protocols were in accordance with the ethical guidelines for Animal Experimentation of the National Research Council of Thailand and were approved by the Animal Ethics Committee of Khon Kaen University (IACUC-KKU-65/65 and IACUC-KKU-52/61). All methods align with the ARRIVE guidelines. Efforts were made to minimize the use of animals and alleviate pain and discomfort.

#### Xenograft mouse model

Six- to eight-week-old female BALB/cAJcl-nu mice were subcutaneously injected with KKU-213B^GemR^cells (1 × 10^6^ cell/each) with Matrigel (1:1) (Corning). Tumors were observed for 21 days until they reached a size of at least 100 mm^3^. Starting on day 22 post-injection, mice were administered 20 mg/kg BW curcumin via oral gavage^49^ once daily, or 50 mg/kg BW gemcitabine via intraperitoneal injection twice a week^54^ or the combination of curcumin and gemcitabine. Tumor size was measured each week with calipers, and mice were euthanized four weeks later. Tumors were collected for immunohistochemistry, western blot, and the tumor volume^54^ and the tumor growth rate^55^ were calculated using the following formulas (1) and (2):

1 $$Tumor\, \text{volume}=\frac{({Width}^{2}\times Length)}{2}$$

2 $$Tumor\, \text{growth rate},\text{ TRG }(\text{\%}) =\frac{(\text{Tumor volume})}{\text{Initial tumor volume}}\times 100$$

#### Hamster model

Four- to six-week-old male golden hamsters were maintained in the experimental animal unit. Twenty-five hamsters were equally divided into 5 groups: normal control, CCA control, CCA hamsters treated with either curcumin alone, gemcitabine alone, or a combination of curcumin and gemcitabine. The development of CCA in hamsters has been described previously^11^. Briefly, CCA was induced by a combination of oral infection with *O. viverrini* (50 metacercariae per hamster) and administration of 12.5 ppm *N*-nitrosodimethylamine (NDMA) (Fujifilm Wako pure chemical corporation, Osaka, Japan) in water for a period of two months. Three months after induction of CCA, hamsters received oral administration of 50 mg/kg curcumin, once daily^56^ and/or intraperitoneal injection of 50 mg/kg gemcitabine, twice a week^54^ for 30 days. After 1 month of treatment, animals were euthanized by overdoses of inhaled anesthetics administered in a chamber using isoflurane. The liver was then collected.

### Histopathology

Sections of hamster livers fixed in 10% formalin and sectioned at 4 µm thickness. The paraffin sections were deparaffinized and rehydrated and stained with hematoxylin and eosin (H&E). The criteria for grading were based on pathology changes in the liver, followed by the International Harmonization of Nomenclature and Diagnostic Criteria for Lesions in Rats and Mice (INHAND)^57^. The entire liver was checked using histopathology, and the foci of the liver lesions, including chronic inflammatory cells, cholangiofibrosis, cholangiocarcinoma, and cholangiofibroma were counted^57,58^. Scores were assigned based on quantifiable findings (number of lesion foci) as follows^57^: grade 0, no foci: grade 1+, 1–2 foci: grade 2+, 3–6 foci: grade 3+, 7–12 foci and, grade 4+, more than 12 foci and grade 5+, diffuse. At least two independent researchers assigned the grading scores in a double-blind manner. The data were represented as relative histopathology change (%).

### Immunohistochemistry

Tissue blocks were sectioned at 4 µm thickness of liver tissue and placed on coated slides. The paraffin sections were deparaffinized and rehydrated. Antigen was retrieved by autoclaving at 110 °C for 10 min in 1X (10 mM) citrate buffer. Endogenous peroxidase activity was blocked with 3% H_2_O_2_ in methanol for 15 min at 25 °C. Nonspecific binding was blocked with 5% FBS in PBS for 30 min. Rabbit anti-CK19 (1:500, ab15463) and rabbit anti-HMGB-1 (1:500, ab79823) from Abcam Inc. in Toronto, ON, Canada, and mouse anti-*SLC7A8* (1:100, GTX83618) from GeneTex in CA, USA, were added to the slides as primary antibodies and incubated overnight. The slides were then incubated with a 1:1000 dilution of HRP-labeled goat anti-rabbit IgG or HRP-conjugated horse anti-mouse IgG (Cell Signaling Technology) IgG for 1h at 25 °C. Immunoreactivity was visualized with 3,3-diaminobenzidine and slides were counterstained with Mayer’s hematoxylin. Slides were dehydrated and visualized under a phase-contrast microscope (Nikon Corporation, Tokyo, Japan).

### Statistics

All data were expressed as mean ± SD and analyzed using GraphPad Prism 9.0. One-way analysis of variance (one-way ANOVA) and Student’s t-test were used to test for differences between experimental groups. To compare among or between experimental groups, significance was set at 0.05.

### Supplementary Information

Supplementary Figures.

Supplementary Table S1.

Supplementary Table S2.

Supplementary Table S3.

Supplementary Table S4.

## Data Availability

The raw RNA sequencing data have been deposited on the Sequencing Reads Archive (SRA) under accession number PRJNA1116859 (https://www.ncbi.nlm.nih.gov/sra/PRJNA1116859). Other datasets used and/or analyzed during the current study available from the corresponding author on reasonable request.

## References

[1] Sarcognato S (2021). Cholangiocarcinoma. Pathologica..

[2] Chaiteerakij R (2017). Characteristics and outcomes of cholangiocarcinoma by region in Thailand: A nationwide study. World J. Gastroenterol..

[3] Vaquero J (2017). Epithelial–mesenchymal transition in cholangiocarcinoma: From clinical evidence to regulatory networks. J. Hepatol..

[4] Khan SA, Tavolari S, Brandi G (2019). Cholangiocarcinoma: Epidemiology and risk factors. Liver Int..

[5] Sirica AE (2005). Cholangiocarcinoma: Molecular targeting strategies for chemoprevention and therapy. Hepatology..

[6] Wongwattanakul M (2017). Classification of Gemcitabine resistant Cholangiocarcinoma cell lines using synchrotron FTIR microspectroscopy. J. Biophotonics..

[7] Giordano, A. & Tommonaro, G. Curcumin and cancer. *Nutrients.* **11** (2019). 10.3390/nu11102376 PMC6835707 31590362

[8] Ming T (2022). Curcumin: An epigenetic regulator and its application in cancer. Biomed. Pharmacother..

[9] Rahmani AH, Alsahli MA, Aly SM, Khan MA, Aldebasi YH (2018). Role of curcumin in disease prevention and treatment. Adv. Biomed. Res..

[10] Prakobwong S (2011). Curcumin suppresses proliferation and induces apoptosis in human biliary cancer cells through modulation of multiple cell signaling pathways. Carcinogenesis..

[11] Prakobwong S (2011). Curcumin decreases cholangiocarcinogenesis in hamsters by suppressing inflammation-mediated molecular events related to multistep carcinogenesis. Int. J. Cancer..

[12] Yoshida K, Toden S, Ravindranathan P, Han H, Goel A (2017). Curcumin sensitizes pancreatic cancer cells to gemcitabine by attenuating PRC2 subunit EZH2, and the lncRNA PVT1 expression. Carcinogenesis..

[13] Cho CJ (2019). The modulation study of multiple drug resistance in bladder cancer by curcumin and resveratrol. Oncol. Lett..

[14] Fan WH, Wang FC, Jin Z, Zhu L, Zhang JX (2022). Curcumin synergizes with cisplatin to inhibit colon cancer through targeting the microRNA-137-glutaminase axis. Curr. Med. Sci..

[15] Coloff JL (2016). Differential glutamate metabolism in proliferating and quiescent mammary epithelial cells. Cell Metab..

[16] Wang Q, Holst J (2015). L-type amino acid transport and cancer: Targeting the mTORC1 pathway to inhibit neoplasia. Am. J. Cancer Res..

[17] El Ansari R (2020). The solute carrier *SLC7A8* is a marker of favourable prognosis in ER-positive low proliferative invasive breast cancer. Breast Cancer Res. Treat..

[18] Xiong G (2019). Long noncoding RNA GSTM3TV2 upregulates LAT2 and OLR1 by competitively sponging let-7 to promote gemcitabine resistance in pancreatic cancer. J. Hematol. Oncol..

[19] Kim, G. W. *et al.* Glutamine synthetase as a therapeutic target for cancer treatment. *Int. J. Mol. Sci.* **22** (2021). 10.3390/ijms22041701 PMC7915753 33567690

[20] Wattanawongdon W (2015). Establishment and characterization of gemcitabine-resistant human cholangiocarcinoma cell lines with multidrug resistance and enhanced invasiveness. Int. J. Oncol..

[21] Yang Y, Li J, Yao L, Wu L (2021). Effect of photodynamic therapy on gemcitabine-resistant cholangiocarcinoma in vitro and in vivo through KLF10 and EGFR. Front. Cell Dev. Biol..

[22] Kunnumakkara AB (2007). Curcumin potentiates antitumor activity of gemcitabine in an orthotopic model of pancreatic cancer through suppression of proliferation, angiogenesis, and inhibition of nuclear factor-kappa B-regulated gene products. Cancer Res..

[23] Liu P (2020). Curcumin enhances anti-cancer efficacy of either gemcitabine or docetaxel on pancreatic cancer cells. Oncol. Rep..

[24] Hu A (2017). Curcumin induces G2/M cell cycle arrest and apoptosis of head and neck squamous cell carcinoma in vitro and in vivo through ATM/Chk2/p53-dependent pathway. Oncotarget..

[25] Weir NM (2007). Curcumin induces G2/M arrest and apoptosis in cisplatin-resistant human ovarian cancer cells by modulating Akt and p38 MAPK. Cancer Biol. Ther..

[26] Chen M (2023). Curcumin analog WZ26 induces ROS and cell death via inhibition of STAT3 in cholangiocarcinoma. Cancer Biol. Ther..

[27] Kahya, U., Koseer, A. S. & Dubrovska, A. Amino acid transporters on the guard of cell genome and epigenome. *Cancers (Basel).* **13** (2021). 10.3390/cancers13010125 PMC7796306 33401748

[28] Feng M (2018). LAT2 regulates glutamine-dependent mTOR activation to promote glycolysis and chemoresistance in pancreatic cancer. J. Exp. Clin. Cancer Res..

[29] Hurkmans EGE (2022). SLC7A8 coding for LAT2 is associated with early disease progression in osteosarcoma and transports doxorubicin. Front. Pharmacol..

[30] Wang FM (2024). SLC7A8 overexpression inhibits the growth and metastasis of lung adenocarcinoma and is correlated with a dismal prognosis. Aging (Albany NY)..

[31] Edemir, B. identification of prognostic organic cation and anion transporters in different cancer entities by in silico analysis. *Int. J. Mol. Sci.* **21** (2020). 10.3390/ijms21124491 PMC7349956 32599841

[32] Xu X (2023). Metabolic reprogramming and epigenetic modifications in cancer: From the impacts and mechanisms to the treatment potential. Exp. Mol. Med..

[33] Dey P, Kimmelman AC, DePinho RA (2021). Metabolic codependencies in the tumor microenvironment. Cancer Discov..

[34] Wang Z (2020). Targeting glutaminolysis: New perspectives to understand cancer development and novel strategies for potential target therapies. Front. Oncol..

[35] Cao J (2019). Expression of GLS1 in intrahepatic cholangiocarcinoma and its clinical significance. Mol. Med. Rep..

[36] Wang Y (2017). GLUL promotes cell proliferation in breast cancer. J. Cell Biochem..

[37] Tardito S (2015). Glutamine synthetase activity fuels nucleotide biosynthesis and supports growth of glutamine-restricted glioblastoma. Nat. Cell Biol..

[38] Shi, X., Zhang, X., Yi, C., Liu, Y. & He, Q. [(1)(3)N]Ammonia positron emission tomographic/computed tomographic imaging targeting glutamine synthetase expression in prostate cancer. *Mol. Imaging.* **13** (2014). 10.2310/7290.2014.00048 25431095

[39] Shao M (2023). Glutamine synthetase-negative hepatocellular carcinoma has better prognosis and response to sorafenib treatment after hepatectomy. Chin. Med. J. (Engl.)..

[40] Duan X, Zhao T, Wang J, Wang J, Zheng Y (2023). Curcumol targets glutaminase 1 to regulate glutamine metabolism and induce senescence of hepatic stellate cells. Eur. J. Integr. Med..

[41] Ni R (2023). Rethinking glutamine metabolism and the regulation of glutamine addiction by oncogenes in cancer. Front. Oncol..

[42] Wang B, Pei J, Xu S, Liu J, Yu J (2024). A glutamine tug-of-war between cancer and immune cells: Recent advances in unraveling the ongoing battle. J. Exp. Clin. Cancer Res..

[43] Kawanishi S, Hiraku Y, Pinlaor S, Ma N (2006). Oxidative and nitrative DNA damage in animals and patients with inflammatory diseases in relation to inflammation-related carcinogenesis. Biol. Chem..

[44] Yongvanit P, Pinlaor S, Bartsch H (2012). Oxidative and nitrative DNA damage: key events in opisthorchiasis-induced carcinogenesis. Parasitol. Int..

[45] Leelawat K (2012). Prognostic relevance of circulating CK19 mRNA in advanced malignant biliary tract diseases. World J. Gastroenterol..

[46] Wang S, Zhang Y (2020). HMGB1 in inflammation and cancer. J. Hematol. Oncol..

[47] Zhuo JY (2020). CK19-positive hepatocellular carcinoma is a characteristic subtype. J. Cancer..

[48] Khoontawad J (2018). Discovering proteins for chemoprevention and chemotherapy by curcumin in liver fluke infection-induced bile duct cancer. PLoS One..

[49] Jantawong C (2023). Curcumin-loaded nanocomplexes alleviate the progression of fluke-related cholangiocarcinoma in hamsters. Cancer Nanotechnol..

[50] Sripa B (2005). Establishment and characterization of an opisthorchiasis-associated cholangiocarcinoma cell line (KKU-100). World J. Gastroenterol..

[51] Sripa B (2020). Functional and genetic characterization of three cell lines derived from a single tumor of an *Opisthorchis viverrini*-associated cholangiocarcinoma patient. Hum. Cell..

[52] Zheng S (2022). SynergyFinder plus: Toward better interpretation and annotation of drug combination screening datasets. Genom. Proteom. Bioinform..

[53] Pinlaor S (2009). Curcumin reduces oxidative and nitrative DNA damage through balancing of oxidant-antioxidant status in hamsters infected with *Opisthorchis viverrini*. Mol. Nutr. Food Res..

[54] Paolino D (2010). Gemcitabine-loaded PEGylated unilamellar liposomes vs GEMZAR: Biodistribution, pharmacokinetic features and in vivo antitumor activity. J. Control Release..

[55] Liu D (2012). Necrosis of cervical carcinoma by dichloroacetate released from electrospun polylactide mats. Biomaterials..

[56] Charoensuk L (2016). Nanoencapsulated curcumin and praziquantel treatment reduces periductal fibrosis and attenuates bile canalicular abnormalities in *Opisthorchis viverrini*-infected hamsters. Nanomedicine..

[57] Thoolen B (2010). Proliferative and nonproliferative lesions of the rat and mouse hepatobiliary system. Toxicol. Pathol..

[58] Narama I (2003). A review of nomenclature and diagnostic criteria for proliferative lesions in the liver of rats by a working group of the Japanese Society of Toxicologic Pathology. J. Toxicol. Pathol..

