# Supplementary material for: Curcumin synergistically enhances the efficacy of gemcitabine against gemcitabine-resistant cholangiocarcinoma via the targeting LAT2/glutamine pathway
Source: Sci Rep. 2024 Jul 11;14:16059. doi: 10.1038/s41598-024-66945-7 (PMC11239878; doi:10.1038/s41598-024-66945-7)
Supplement: Supplementary file 1 — Supplementary Figures. [file 41598_2024_66945_MOESM1_ESM.docx]

**Supplementary data**

**Curcumin synergistically enhances the efficacy of gemcitabine against gemcitabine-resistant cholangiocarcinoma via the** **targeting LAT2/glutamine pathway**

Phonpilas Thongpon^a,g^, Kitti Intuyod^b,g^, Sasitorn Chomwong^a,g^, Thatsanapong Pongking^c,g^, Sirinapha Klungsaeng^a,g^, Kanha Muisuk^d^, Naruechar Charoenram^a,g^, Chutima Sitthirach^a,g,^  Raynoo Thanan^e,g^, Porntip Pinlaor^f,g^, Somchai Pinlaor ^a,g^*

*^a^Department of Parasitology, Faculty of Medicine, Khon Kaen University, Khon Kaen 40002, Thailand*

*^b^Department of Pathology, Faculty of Medicine, Khon Kaen University, Khon Kaen 40002, Thailand*

*^c^Biomedical Sciences Program, Graduate School, Khon Kaen University, Khon Kaen 40002, Thailand*

*^d^Department of Forensic Medicine, Faculty of Medicine, Khon Kaen University, Khon Kaen 40002, Thailand*

*^e^Department of Biochemistry, Faculty of Medicine, Khon Kaen University, Khon Kaen 40002, Thailand*

*^f^Centre for Research and Development in Medical Diagnostic Laboratory, Faculty of Associated Medical Sciences, Khon Kaen University, Khon Kaen 40002, Thailand*

*^g^Cholangiocarcinoma Research Institute, Khon Kaen University, Khon Kaen 40002, Thailand.*

**Corresponding author*

*Prof. Somchai Pinlaor, Department of Parasitology, Faculty of Medicine, Khon Kaen University, Khon Kaen 40002, Thailand. Tel: +66 895752800 E-mail address: psomec@kku.ac.th*

**Legend**

**
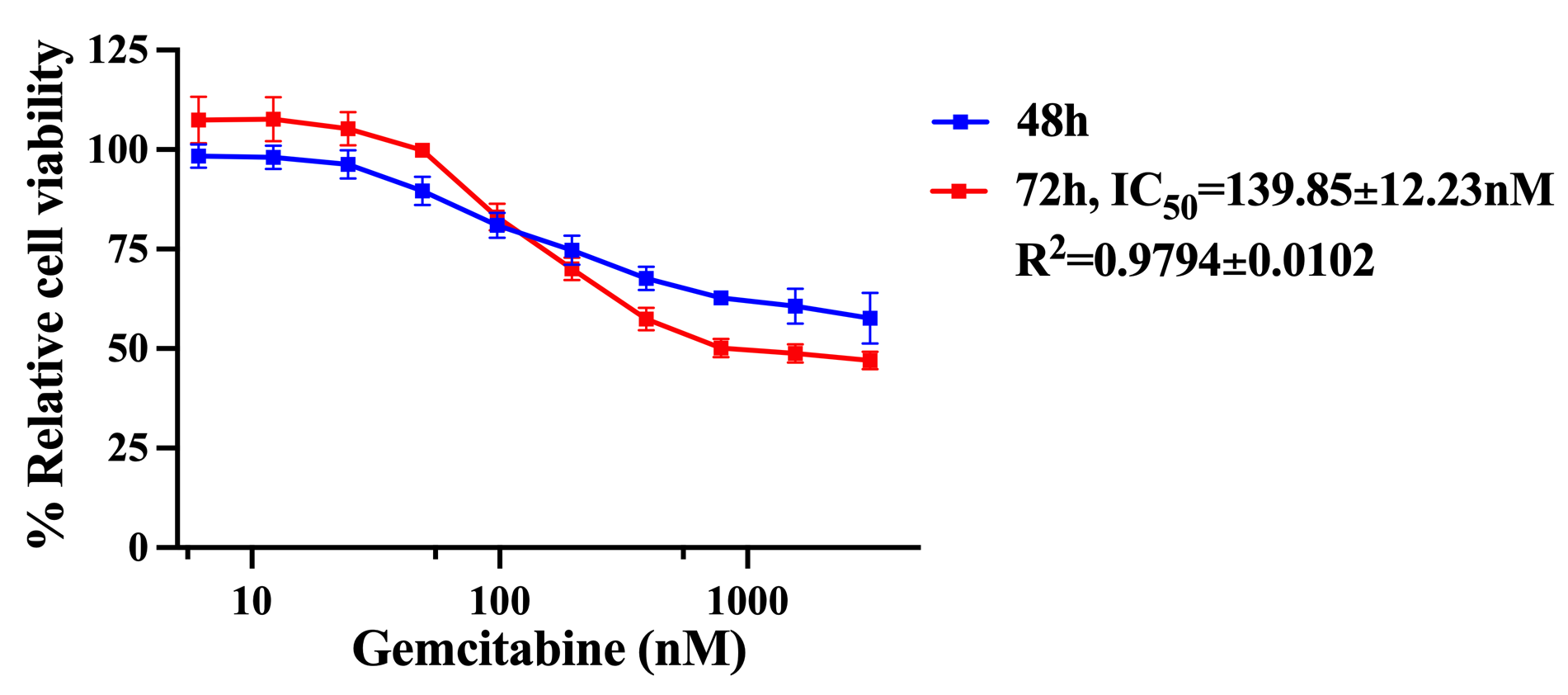
**

**Supplementary Fig. S1** The effect of gemcitabine against resistant CCA cell line (KKU-213B^GemR^) at 40 cycle induction using the MTT assay (48-72h). All data were expressed as the mean ± SD from three replicates of two independent experiments.

**
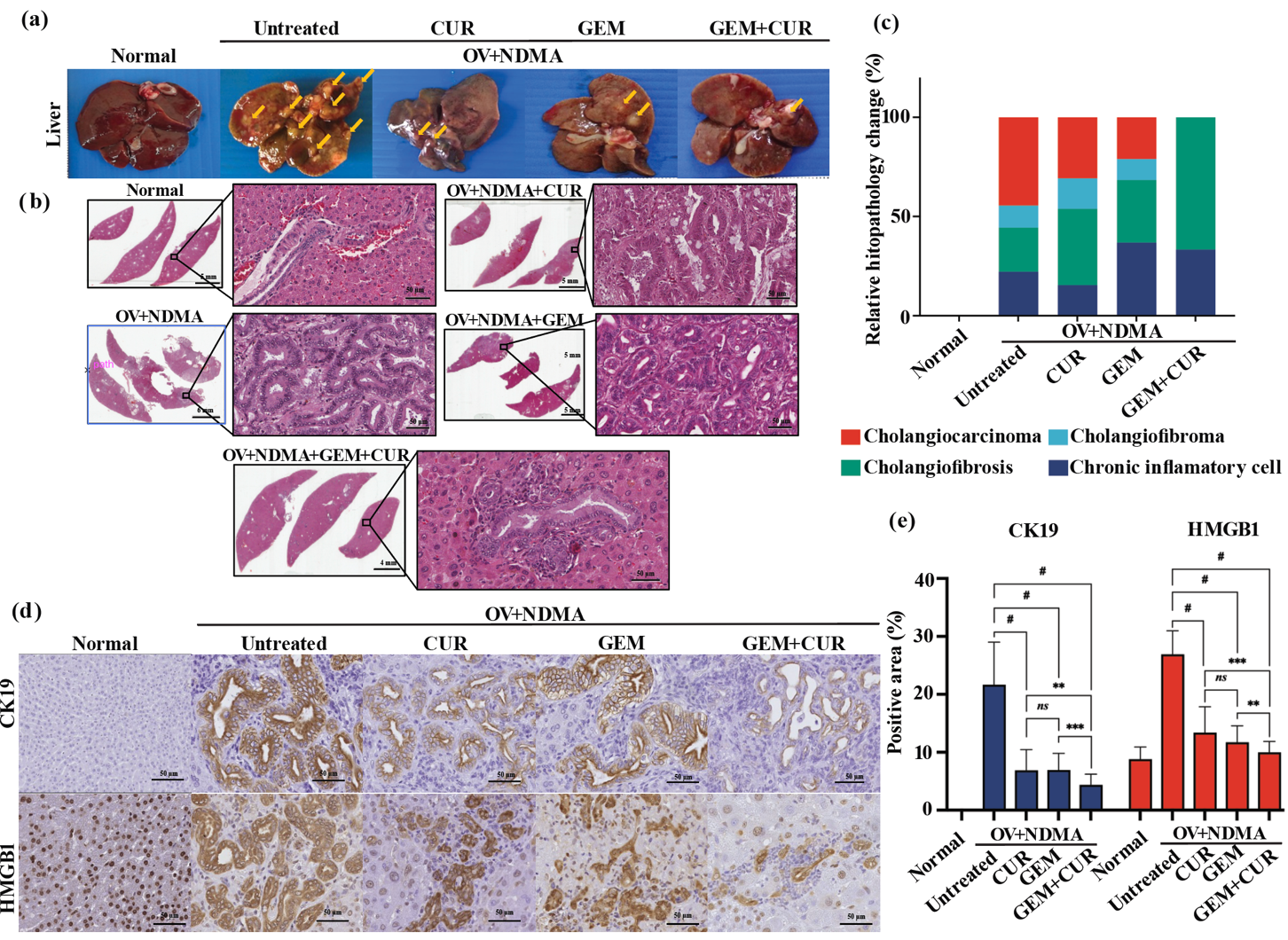
Supplementary Fig. S2** Anti-tumor effects of curcumin, gemcitabine and their combination in an *O. viverrini*-associated CCA in an orthotopic hamster model. (a) Liver gross and tumor mass/tumor fibrosis (yellow arrows), (b) H&E staining and immunohistochemical assay and (c) the relative histhopatology change (%) of hamster livers. (d+e) The positive-staining areas of CK19 and HMGB1. All data are expressed as means ± SD of three biological independent experiments; *ns=*not significant, ** *p*<0.01, ****p<*0.001 and #*p*<0.0001 compared between groups (N= 1-3): Curcumin, GEM: Gemcitabine
