# Supplementary material for: Curcumin synergistically enhances the efficacy of gemcitabine against gemcitabine-resistant cholangiocarcinoma via the targeting LAT2/glutamine pathway
Source: Sci Rep. 2024 Jul 11;14:16059. doi: 10.1038/s41598-024-66945-7 (PMC11239878; doi:10.1038/s41598-024-66945-7)
Supplement: Supplementary file 2 — Supplementary Table S1. [file 41598_2024_66945_MOESM2_ESM.docx]

**Supplementary data**

**Curcumin synergistically enhances the efficacy of gemcitabine against gemcitabine-resistant cholangiocarcinoma via the** **targeting LAT2/glutamine pathway**

Phonpilas Thongpon^a,g^, Kitti Intuyod^b,g^, Sasitorn Chomwong^a,g^, Thatsanapong Pongking^c,g^, Sirinapha Klungsaeng^a,g^, Kanha Muisuk^d^, Naruechar Charoenram^a,g^, Chutima Sitthirach^a,g,^  Raynoo Thanan^e,g^, Porntip Pinlaor^f,g^, Somchai Pinlaor ^a,g^*

*^a^Department of Parasitology, Faculty of Medicine, Khon Kaen University, Khon Kaen 40002, Thailand*

*^b^Department of Pathology, Faculty of Medicine, Khon Kaen University, Khon Kaen 40002, Thailand*

*^c^Biomedical Sciences Program, Graduate School, Khon Kaen University, Khon Kaen 40002, Thailand*

*^d^Department of Forensic Medicine, Faculty of Medicine, Khon Kaen University, Khon Kaen 40002, Thailand*

*^e^Department of Biochemistry, Faculty of Medicine, Khon Kaen University, Khon Kaen 40002, Thailand*

*^f^Centre for Research and Development in Medical Diagnostic Laboratory, Faculty of Associated Medical Sciences, Khon Kaen University, Khon Kaen 40002, Thailand*

*^g^Cholangiocarcinoma Research Institute, Khon Kaen University, Khon Kaen 40002, Thailand.*

**Corresponding author*

*Prof. Somchai Pinlaor, Department of Parasitology, Faculty of Medicine, Khon Kaen University, Khon Kaen 40002, Thailand. Tel: +66 895752800 E-mail address: psomec@kku.ac.th*

**Supplementary Table S1.** Comparison of STR profiles of CCA cell line (KKU-213B) and gemcitabine-resistant CCA cell line (KKU-213B^GemR^).

| **Loci** | **Autosomal STR profiles** | | **Loci** | **X-STR profiles** | |
| --- | --- | --- | --- | --- | --- |
|  | KKU-213B | KKU-213B^GemR^ |  | KKU-213B | KKU-213B^GemR^ |
| D3S1358 | 15, 15 | 15, 15 | DXS10103 | 19, 19 | 19, 19 |
| vWA | 18, 19 | 18, 19 | DXS8378 | 11, 11 | 11, 11 |
| D16S539 | 9, 11 | 9, 11 | DXS10101 | 31, 31 | 31, 31 |
| CSF1PO | 9,13 | 9,13 | DXS10134 | 32, 32 | 32, 32 |
| D6S1043 | 12, 15 | 12, 15 | DXS10074 | 19, 19 | 19, 19 |
| D8S1179 | 11, 13 | 11, 13 | DXS7132 | 15, 15 | 15, 15 |
| D21S11 | 29, 29 | 29, 29 | DXS10135 | 27, 27 | 27, 27 |
| D18S51 | 14, 19 | 14, 19 | DXS7423 | 15, 15 | 15, 15 |
| D5S818 | 9, 9 | 9, 9 | DXS10146 | 26, 26 | 26, 26 |
| D2S441 | 10, 10 | 10, 10 | DXS10079 | 19, 20 | 20, 20 |
| D19S433 | 13, 14 | 13, 14 | HPRTB | 11, 12 | 11, 12 |
| FGA | 16, 16 | 16, 16 | DXS10148 | 22.1, 22.1 | 22.1, 22.1 |
| D10S1248 | 14, 15 | 14, 15 | D21S11 | 29, 29 | 29, 29 |
| D22S1045 | 15, 15 | 15, 15 | Amelogenin | X | X |
| D1S1656 | 14, 15 | 14, 15 |  |  |  |
| D13S317 | 8, 12 | 8, 12 |  |  |  |
| D7S820 | 11, 11 | 11, 11 |  |  |  |
| Penta E | 11, 13 | 11, 13 |  |  |  |
