# Supplementary material for: Curcumin synergistically enhances the efficacy of gemcitabine against gemcitabine-resistant cholangiocarcinoma via the targeting LAT2/glutamine pathway
Source: Sci Rep. 2024 Jul 11;14:16059. doi: 10.1038/s41598-024-66945-7 (PMC11239878; doi:10.1038/s41598-024-66945-7)
Supplement: Supplementary file 4 — Supplementary Table S3. [file 41598_2024_66945_MOESM4_ESM.docx]

**Supplementary data**

**Curcumin synergistically enhances the efficacy of gemcitabine against gemcitabine-resistant cholangiocarcinoma via the** **targeting LAT2/glutamine pathway**

Phonpilas Thongpon^a,g^, Kitti Intuyod^b,g^, Sasitorn Chomwong^a,g^, Thatsanapong Pongking^c,g^, Sirinapha Klungsaeng^a,g^, Kanha Muisuk^d^, Naruechar Charoenram^a,g^, Chutima Sitthirach^a,g,^  Raynoo Thanan^e,g^, Porntip Pinlaor^f,g^, Somchai Pinlaor ^a,g^*

*^a^Department of Parasitology, Faculty of Medicine, Khon Kaen University, Khon Kaen 40002, Thailand*

*^b^Department of Pathology, Faculty of Medicine, Khon Kaen University, Khon Kaen 40002, Thailand*

*^c^Biomedical Sciences Program, Graduate School, Khon Kaen University, Khon Kaen 40002, Thailand*

*^d^Department of Forensic Medicine, Faculty of Medicine, Khon Kaen University, Khon Kaen 40002, Thailand*

*^e^Department of Biochemistry, Faculty of Medicine, Khon Kaen University, Khon Kaen 40002, Thailand*

*^f^Centre for Research and Development in Medical Diagnostic Laboratory, Faculty of Associated Medical Sciences, Khon Kaen University, Khon Kaen 40002, Thailand*

*^g^Cholangiocarcinoma Research Institute, Khon Kaen University, Khon Kaen 40002, Thailand.*

**Corresponding author*

*Prof. Somchai Pinlaor, Department of Parasitology, Faculty of Medicine, Khon Kaen University, Khon Kaen 40002, Thailand. Tel: +66 895752800 E-mail address: psomec@kku.ac.th*

**Supplementary Table S3**. Original blotted membranes from KKU-213B^GemR^ cell lines knockdown of *SLC7A8* (LAT2).

| Cell lines | Protein target | Original membranes of target protein | GAPDH |
| --- | --- | --- | --- |
| **KKU-213B^GemR^ with different conditions:**  Control,  Lipofectsmine,  siNon-Target,  si*SLC7A8* | LAT2 | 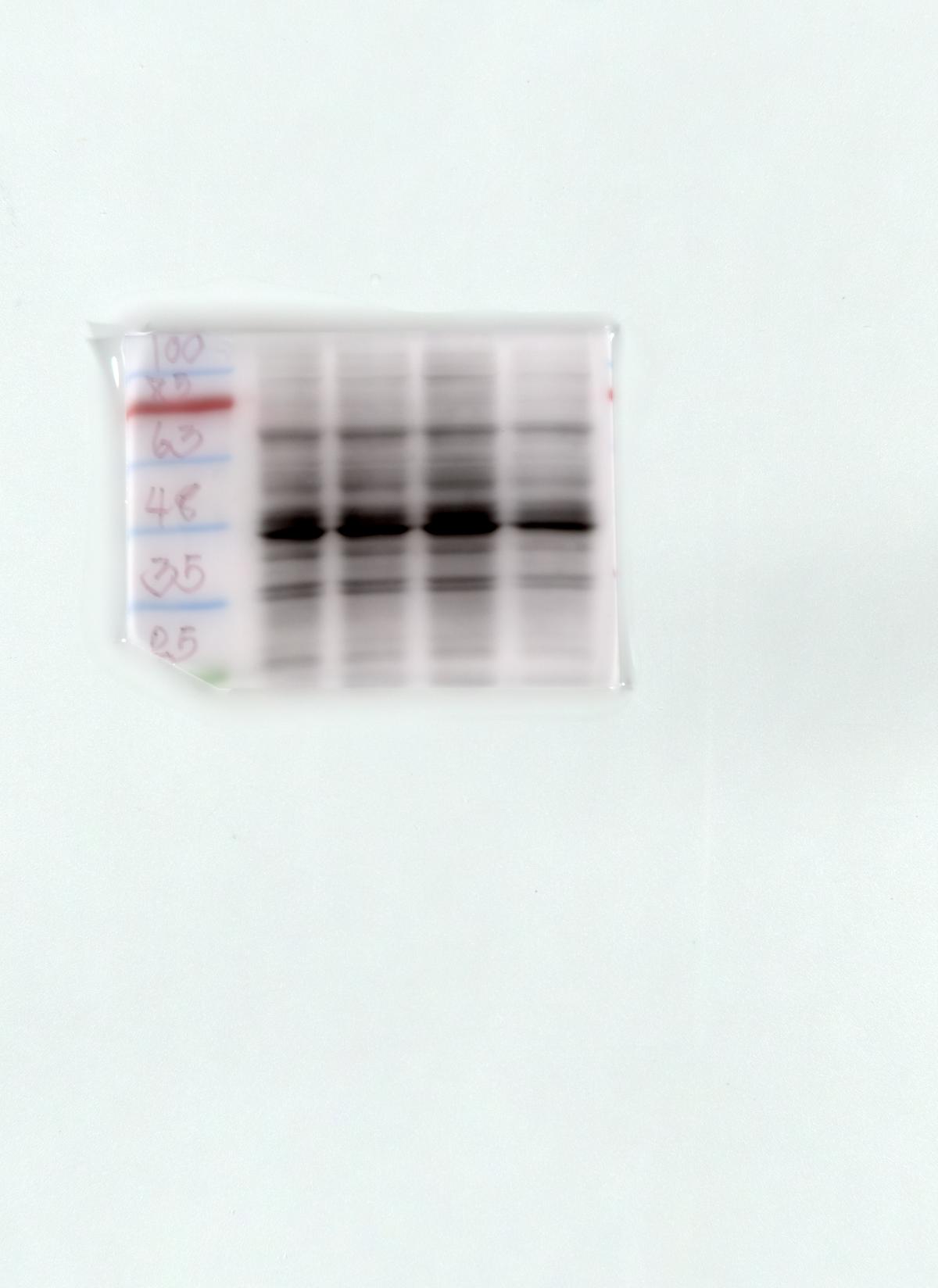  Fig. 6a | 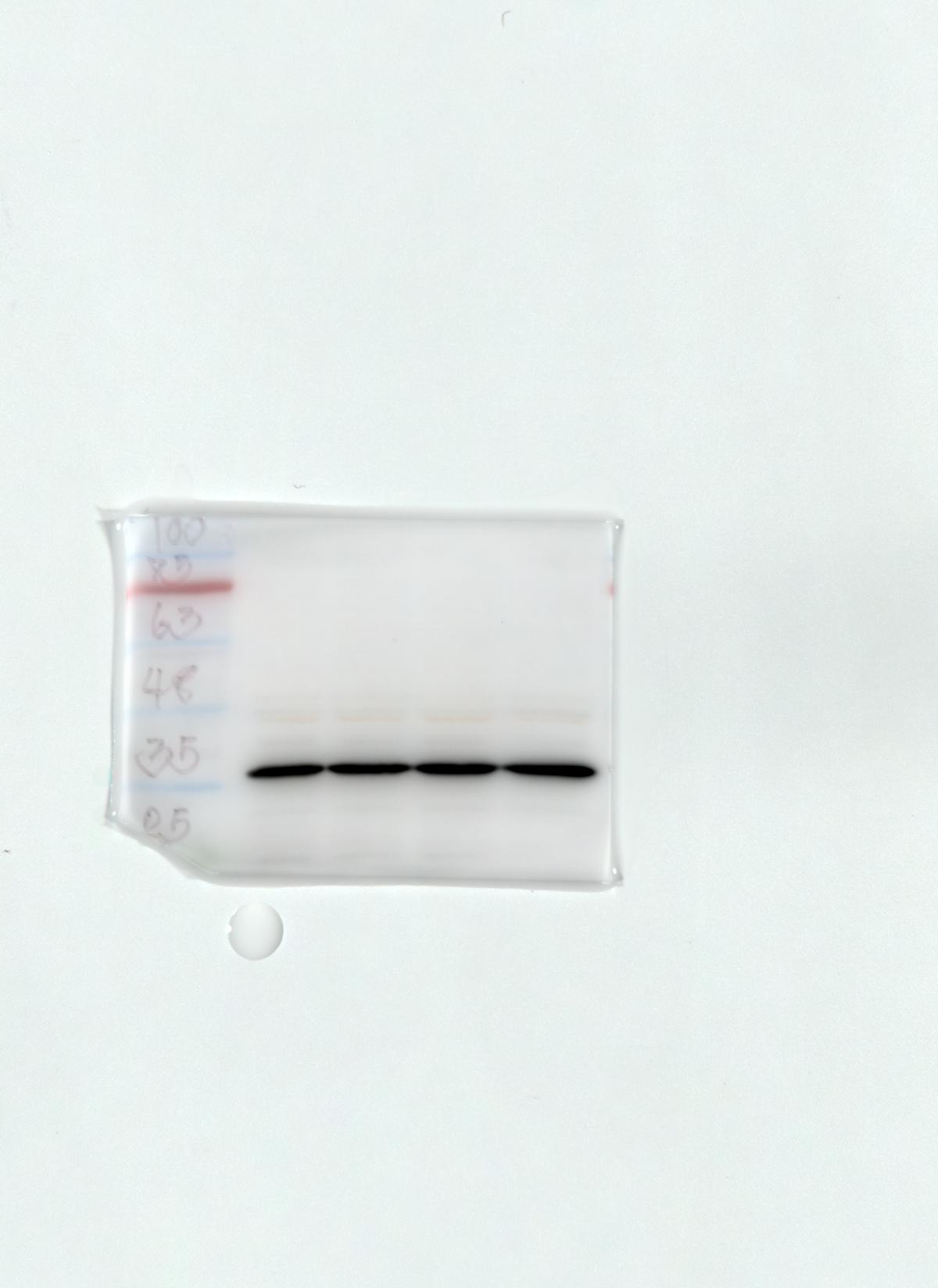  Fig. 6a |
| **KKU-213B^GemR^ with different conditions:**  Control,  Lipofectsmine,  siNon-Target,  si*SLC7A8* | GLS | 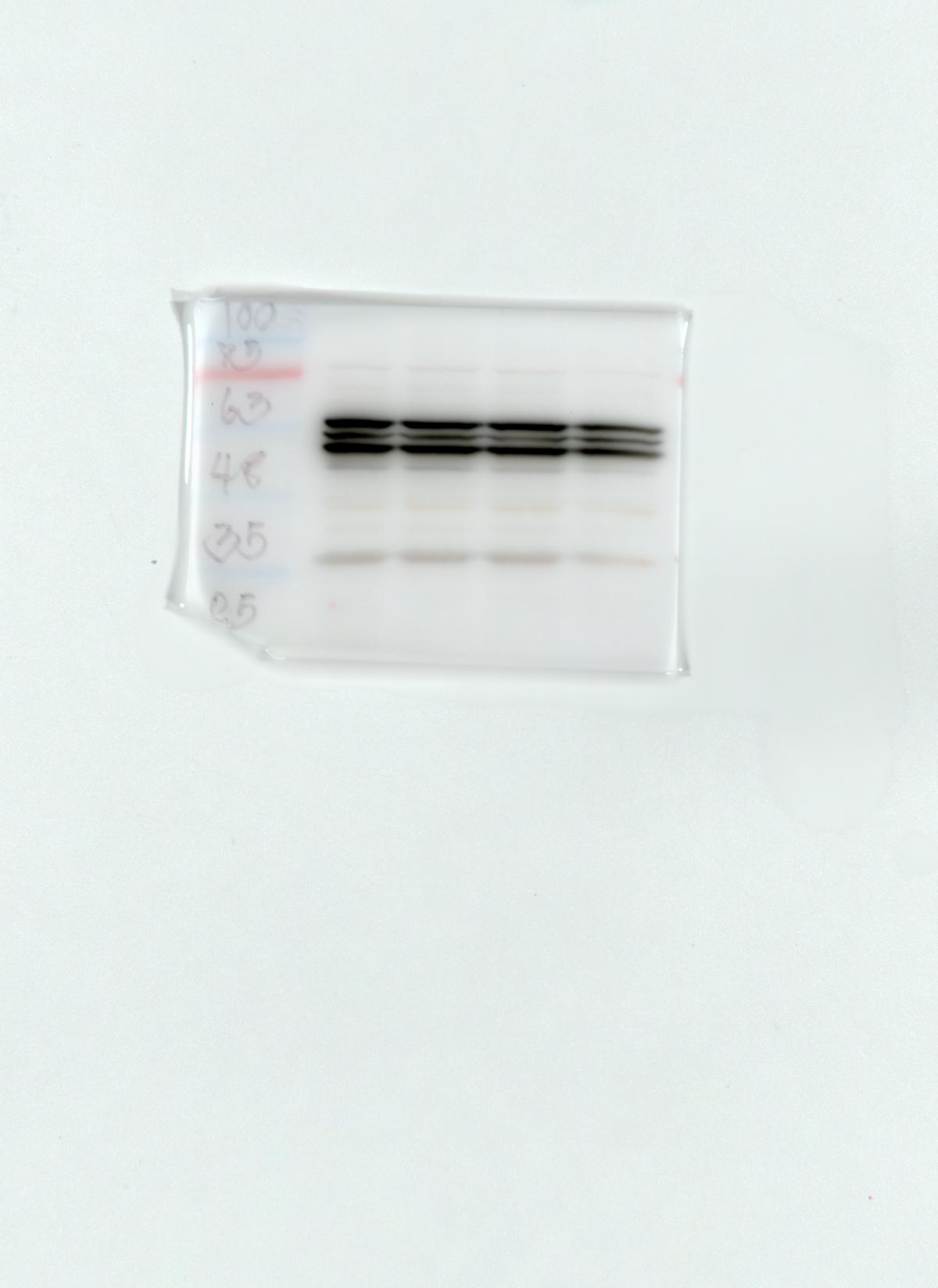  Fig. 6a | 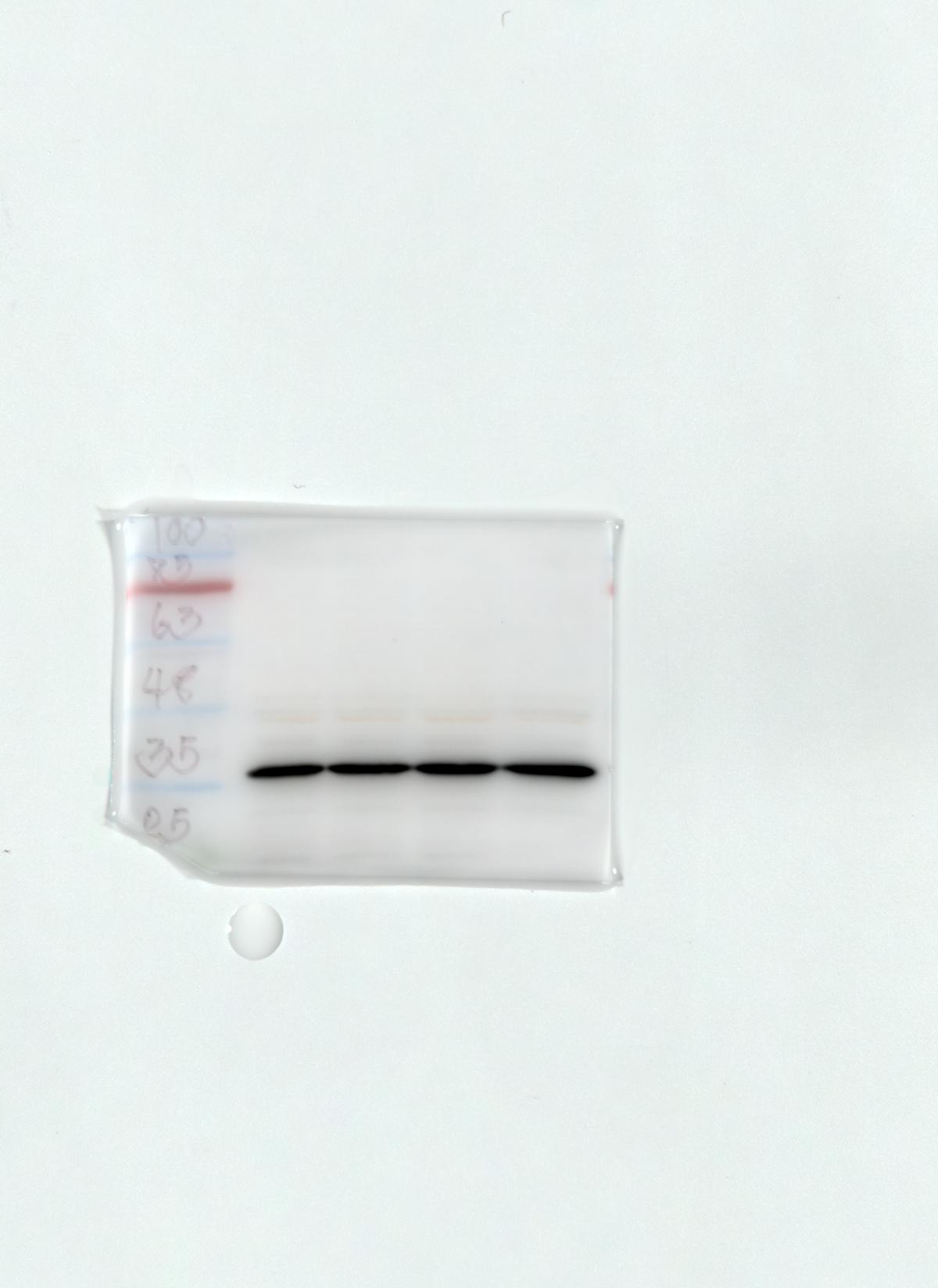  Fig. 6a |
| **KKU-213B^GemR^ with different conditions:**  Control,  Lipofectsmine,  siNon-Target,  si*SLC7A8* | GS | 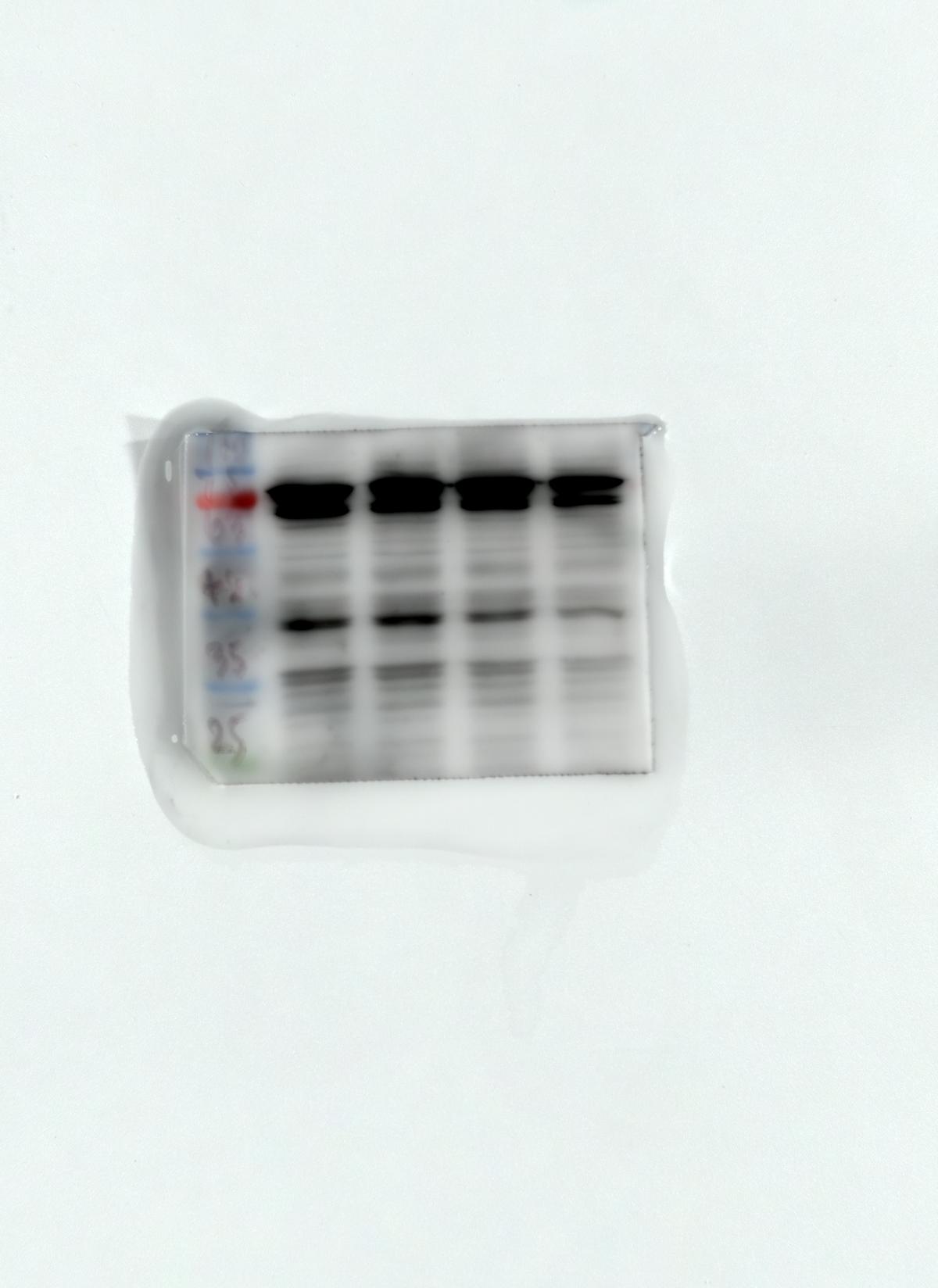  Fig. 6a | 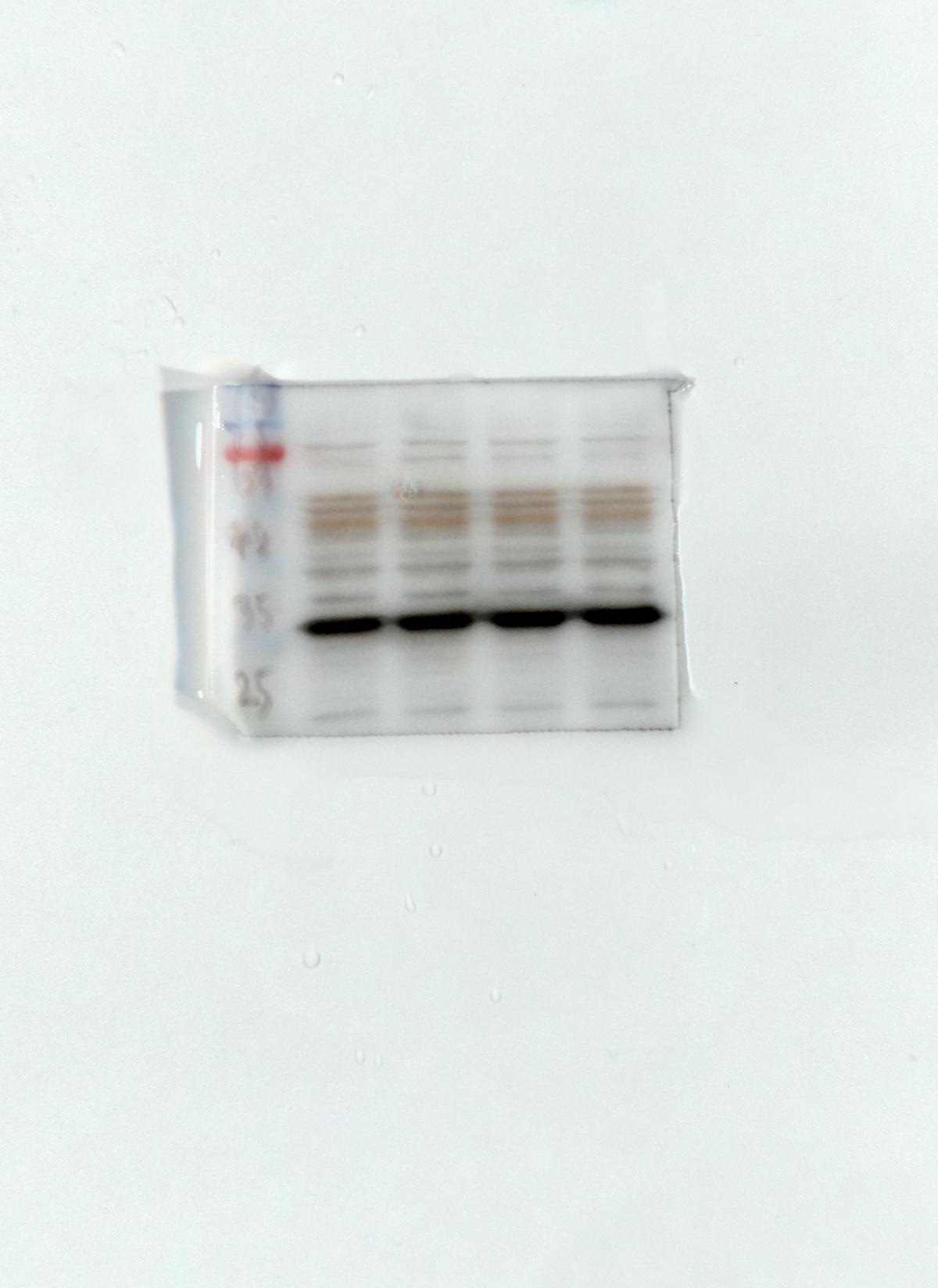  Fig. 6a |
